# Exploring the influence of testimonial source on attitudes towards e-mental health interventions among university students: Four-group randomized controlled trial

**DOI:** 10.1371/journal.pone.0252012

**Published:** 2021-05-26

**Authors:** Jennifer Apolinário-Hagen, Mathias Harrer, Melina Dederichs, Lara Fritsche, Jeannette Wopperer, Frank Wals, Adrian Loerbroks, Dirk Lehr, Christel Salewski, Peter Angerer, David Daniel Ebert

**Affiliations:** 1 Faculty of Medicine, Institute of Occupational, Social and Environmental Medicine, Centre for Health and Society, Heinrich Heine University Düsseldorf, Düsseldorf, Germany; 2 Department of Clinical Psychology and Psychotherapy, Friedrich Alexander University Erlangen-Nuremberg, Erlangen, Germany; 3 Faculty of Psychology, Department of Health Psychology, University of Hagen, Hagen, Germany; 4 Department of Health Psychology, Leuphana University Lueneburg, Lueneburg, Germany; 5 Department of Clinical, Neuro- and Developmental Psychology, Vrije Universiteit Amsterdam, Amsterdam, the Netherlands; University of Macau, MACAO

## Abstract

Electronic mental health services (eMHSs) offer additional options for the dissemination of psychological interventions for university students. Still, many university students are reluctant to use eMHSs. Narrative messages may help increase the awareness and acceptance of quality-approved programs. However, little is known about the usefulness of narrative messages to improve attitudes towards eMHSs. In this experiment, we thus aimed to explore in how far different ways of targeting information to students affect their attitudes towards eMHSs for stress prevention and therapy, and to identify potential determinants of attitude change. N = 451 students (Mean = 32.6 years, SD = 10.2, 75% female, 7% with eMHS experience) were randomly assigned to one of four study arms involving information designed to induce different levels of perceived similarity. While the active control condition only received general information (arm 1, “information only”, n = 116), the other experimental arms were additionally exposed to testimonials on specific eMHSs either addressing an unspecified audience (arm 2, n = 112), employees (arm 3, n = 115) or working university students (arm 4, n = 108). Two-way ANOVA revealed no impact of information on the alteration of attitudes towards eMHSs for stress coping (*d* = 0.20). Only a small effect of target-group specific testimonials on attitudes towards online therapies was identified at post-intervention (*d* = 0.29). Regression analyses demonstrated significant influences of source credibility and perceived similarity on attitudes for preventative eMHSs (*p*_s_<0.01), as well as a partial mediation effect of perceived similarity in favor of testimonials targeted to students (95% CI [0.22, 0.50]). Overall, this study indicated no meaningful impact of information on attitudes and limited evidence for benefits of tailored narrative messages. Since attitudes were already positive at baseline, further research with a representative student sample mimicking real-world decision scenarios is needed to gain an in-depth understanding of acceptance-facilitating message features that may contribute to promote the adoption of evidence-based eMHSs.

## Introduction

In recent years, mental health problems have been recognized as a common risk factor for the academic functioning of college and university students (hereafter referred to as university students) [1]. Mental disorders are relatively prevalent even among freshmen worldwide, with an accumulated 12-month prevalence of 31% [2]. On the one hand, international WHO surveys document a large treatment gap in concerned university students [3]. Besides suboptimal levels of mental health literacy [4], attitudinal barriers to utilize traditional (face-to-face) mental health services such as a preference to manage problems on one’s own, desire for self-reliance or the fear of stigmatization, have been frequently observed among students, and appear to be more problematic than structural obstacles, such as limited access and costs [5–7]. On the other hand, recent research indicates that effective stress prevention could reduce up to 80% of the 12-month prevalence for mental disorders among university students [8].

Electronic mental health services (eMHSs) like stress management apps have been suggested as a promising approach to increase the uptake of interventions for mental health promotion, prevention and self-help therapy by university students on campus [5] and distance-learning students [9]. These services may facilitate the search for professional support and offer greater flexibility, anonymity and accessibility of psychological interventions [10,11]. Although a large body of evidence supports the efficacy of eMHSs in reducing perceived stress and the symptoms of mental health problems among various populations [12] such as university students [13,14], their utilization still remains low [15,16]. So far, prior research indicates ambivalent public attitudes towards eMHSs, low awareness and little experience with such offers in many countries like Germany [16–18], even among future healthcare professionals and digital natives like medical and psychology students [19]. Consequently, raising the awareness of evidence-based eMHSs could be a starting point to promote informed decisions and their uptake by relevant target groups [20,21].

Still, it remains unclear how information on eMHSs should be designed to meet the preferences and needs of both unconcerned and distressed students. One strategy to raise the awareness of eMHSs is providing information supplemented with first-person testimonials. Personal experiences or user reviews are commonly added to descriptions of specific eMHSs (e.g., in app stores [22]). Peer testimonials have also been applied in eMHSs for stress management as an option to tailor intervention elements to students (e.g., [23,24]). Testimonials may be an especially suitable way to provide information for populations without personal experience regarding specific mental health services [25]. As an everyday form of health communication, narrative messages such as first-person testimonials are easy to understand, since they do not require knowledge or personal involvement [26]. According to dual-processing models like the *Elaboration Likelihood Model* (ELM; [27]), persuasion and the persistence of subsequent attitude change depend on whether an individual is more likely to process a message heuristically via the *peripheral route* (e.g., non-message cues like similarity or reputation in testimonial sources) or systematically via the *central route* (e.g., argument strength, using statistics) [26,28,29]. While health communication addressing the *central route* requires both sufficient motivation and ability to process the message, attitudes shaped via the *peripheral route* are oftentimes based on heuristics like “experts know it best” and simple cues (e.g., doctors’ white coat) [30]. Affect can also function as such a peripheral information cue [30]. Regarding eMHSs, perceived stress can signalize the need for psychological support [21] and may contribute to momentary shifts in help-seeking attitudes [16,18,31]. Consequently, using testimonials may be a simple, suitable option to increase the acceptance of eMHSs among students.

To date, however, most research on the effects of health-related testimonials has focused on patient narratives in decision aids and hypothetical medical treatment or screening choices by patients [32]. Much less is known about how unconcerned people process health testimonials with respect to preventative purposes [33]. More remarkably, very few studies have investigated the effects of testimonials on views about psychological interventions such as eMHSs, and the existing research yielded indecisive findings [34–37]. Hence, it might be worthwhile to focus on heuristics related to perceived similarity as well as source credibility in the design of testimonials, which were demonstrated to be persuasive factors across different health communication fields [28]. Yet, it remains unclear whether research evidence from other health communication contexts is also transferable to testimonials on eMHSs. Based on the outlined literature, it can be assumed that testimonials might help arouse initial interest target groups without personal experience with eMHSs, which could later result in more elaborated judgments, but more research is required.

Taken together, the common practice of using testimonials for advertising or tailoring eMHSs to specific target groups is at best supported by limited or coincidental empirical evidence. Specifically, little is known about the added value of testimonials as a supplement to eMHS information varying in the degree of perceived similarity (e.g., testimonials targeted students versus employees vs. unspecified audiences). In this context, it also appears plausible to differentiate between the effects of information on alterations of attitudes towards eMHSs for mental health promotion versus treatment. Possibly, attitudes may be more critical towards digital interventions for dealing with serious mental health problems than for prevention or stress management purposes in healthy people [18,38]. However, it is unclear whether acceptance-facilitating interventions may be more potent to improve rather negative attitudes towards online therapies compared to eMHSs for mental health promotion, or if they are harder to change. In addition, the role of potential mediators like perceived similarity in attitudes after exposure to targeted testimonials on eMHSs requires further investigation. Overall, this knowledge could help optimize the design of information materials aiming at improving the awareness, attitudes and acceptance of eMHSs among university students.

### Objectives

The aim of this experimental study was to determine whether providing different types of information varying in the degree of targeting to students influences attitudes towards eMHSs for stress coping and online therapies among university students. Another purpose was to identify determinants of attitude change and mediating effects of perceived similarity in targeted testimonials.

#### Primary outcome

*Hypothesis 1 (H1)*. Based on prior research, we expected improvements in attitudes towards eMHSs for stress coping (in terms of perceived usefulness) among university students after exposure to information either with or without added testimonials compared to attitudes at the baseline assessment.

#### Secondary outcomes

*Hypothesis 2 (H2)*. Furthermore, we expected a positive attitude change to be more likely when information additionally includes testimonials from sources perceived as similar to oneself. Particularly, we expected more pronounced improvements in attitudes towards eMHSs for stress coping after exposure to information plus testimonials from university students compared to testimonials by employees, untargeted testimonials and information only.

*H2-Sub-hypotheses and research questions*. Since we were interested in comparing attitudes towards eMHSs for prevention or mental health promotion versus treatment, we explored differences between the information groups in (H2a) attitudes towards eMHSs for stress coping (perceived usefulness) and (H2b) multi-facet attitudes towards professionally guided online therapies. As the presented information scoped mainly on mental health promotion, we assessed whether the narrative information on eMHSs for stress coping would also be associated with more positive attitudes towards online therapies at post-intervention (in the sense of transfer effects). We expected a positive influence of information on attitudes regarding both application fields (H2a and H2b). In addition, we explored whether the influence of testimonials will be stronger for eMHSs for stress coping compared to online therapies (H2-related research question).

*Hypothesis 3 (H3)*. Additionally, we assumed an influence of information varying in the degree of targeting to students (targeted to students vs. employees and undefined populations), perceived stress, perceived similarity and source credibility on students’ attitudes towards eMHSs for stress coping at post-intervention, which remains after controlling for the influence of attitudes at baseline.

*Hypothesis 4 (H4)*. Finally, we expected that perceived similarity with presented testimonial sources (university students versus employees) mediates the influence of targeted testimonials on attitudes towards (H4a) eMHSs for stress coping and (H4b) online therapies.

## Materials and methods

### Trial design

The presented four-arm parallel group RCT was anonymously conducted using the German version of the *Unipark* online survey software (Enterprise Feedback Suite (EFS) Survey, Questback). The 2x4 design of this cross-sectional trial employed two time points (pre- and post-intervention assessment) and four experimental study arms. We followed the recommendations of CONSORT extensions for the reporting of psychological experiments, such as CONSORT-PSI 2018 [39–41]. The study was conducted at the University of Hagen (*FernUniversität in Hagen*), which is the largest and only state-hold distance-learning university in Germany with approximately 73.000 enrolled students, including more than 14,000 psychology students [42]. Besides the recommendations of the German Psychological Association [43], our study adheres to the German Federal General Data Protection Act, the Data Protection Regulation and the Declaration of Helsinki in the latest version. Due to the interim phase of the newly established ethic committee of the Faculty of Psychology at the University of Hagen in fall 2018, we were unable to seek formal ethical approval from the Institutional Review Board (IRB). For specific cases, an ad-hoc committee reviewed research studies after consultation with an ethic commissioner of the rectorate of the University of Hagen until the constitution of the IRB in April 2019. In our case, the study received an exemption from ethical approval by the ethic commissioner of the rectorate prior to data collection, since this pre-tested study [35] with a non-clinical scope did not involve ethically sensitive material or burdensome procedures, used approved online survey templates and followed the applicable regulations and ethical guidelines for psychological research. Due the new affiliation of the principal investigator, the ethic committee of the Heinrich Heine University Düsseldorf, Faculty of Medicine, was additionally consulted and responded on November 21, 2019, that they would have approved the study if the proposal had been submitted prior to data collection. This study was not pre-registered, but the reported results were based on pre-defined hypotheses that were presented in short reports by the involved postgraduate students within an experimental M.Sc. Psychology module and at two international conferences [44,45]. This study also involved the investigation of acceptance of and registration rates for eMHSs, but these outcomes will be analyzed and reported elsewhere in due length. Data sets, supplementary output files and original study materials (e.g., online questionnaire) have been archived in *SowiDataNet|datorium* and will be shared openly for research purposes (access via https://doi.org/10.7802/2127).

### Participants

We included German-speaking students enrolled in a college or university who were aged 18 years or older and gave informed consent. Exclusion criteria involved decline or withdrawal of consent (i.e., explicitly per click after debriefing or indirectly via non-completion). Participants were recruited using non-probability convenience sampling from November 27, 2018 until May 15, 2019 via social media websites (e.g., *Facebook*, *Xing*), the eLearning platform *moodle* and the virtual lab of the Faculty of Psychology at the University of Hagen, personal invitation by psychology students and websites with sections for participant recruitment for psychological surveys (*Psychologie Heute* [Psychology Today], and *SurveyCircle*). Participants could receive a summary of aggregated key findings on request. Psychology students could be compensated with course credits via the virtual lab of the faculty, which makes no connection to data sets to ensure anonymity of participants. A priori power analyses using G*Power, version 3.1.9.2 [46] were performed for the primary outcome in order to identify a small effect (*f* = 0.10) in two-way ANOVA (alpha = 0.05, power = 0.95) and yielded a minimum sample size of n = 436. The small effect size was grounded on similar research (e.g., [5,35]).

### Procedure

**Fig 1:**
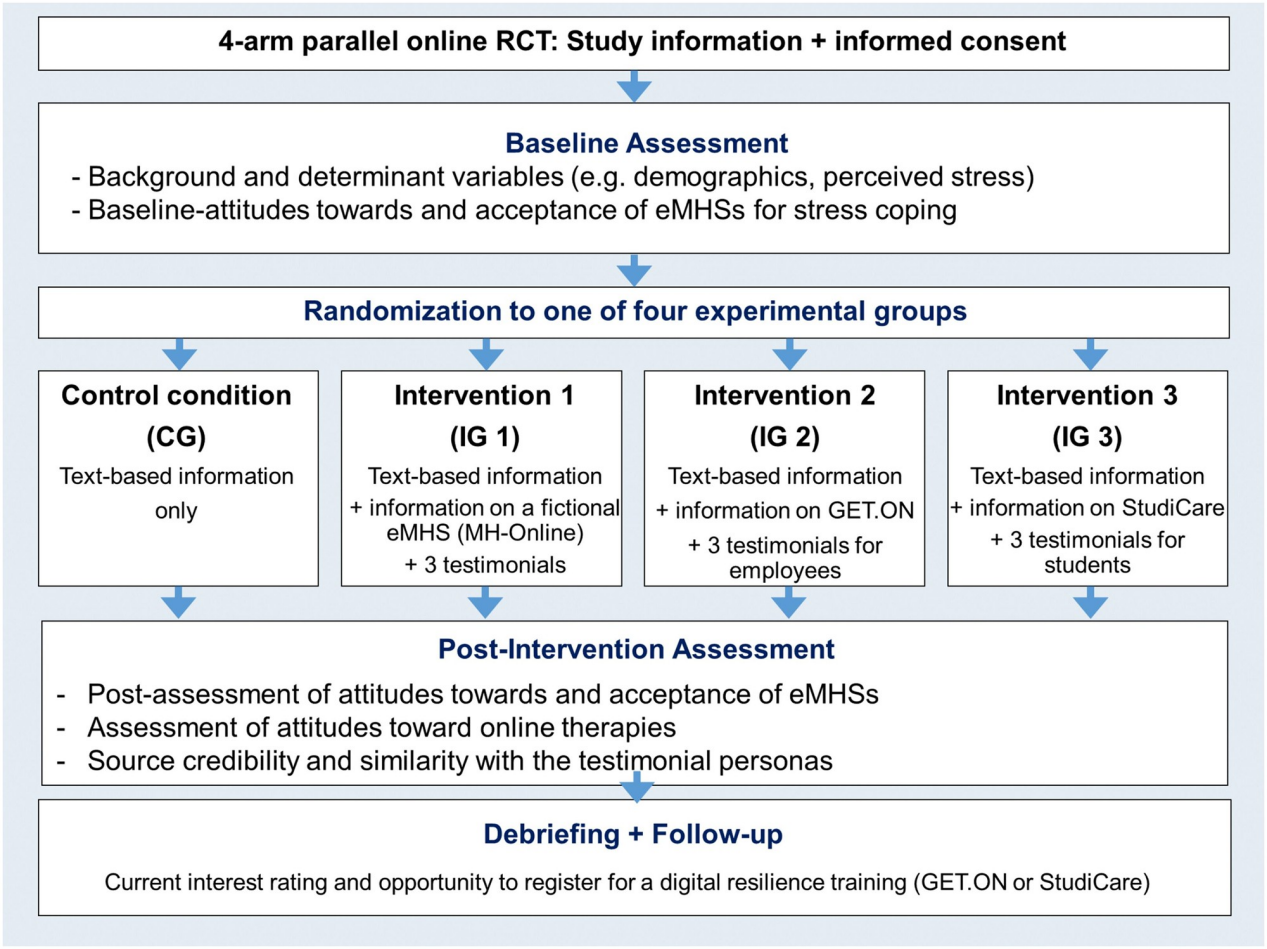
Study procedure of the online experiment. Details on the acceptance outcome and current interest as well as registration rates for two resilience trainings are reported in full length elsewhere.

Following the study information that included a working definition of guided eMHSs and the informed consent (“click to agree”), participants were screened for inclusion criteria. As illustrated in Fig 1, participants were then asked to answer questions on demographic background, awareness of and experience with eMHSs, perceived stress and baseline attitude towards eMHSs (as well as intentions to use, reported elsewhere [45]).

Next, participants were informed about the randomization procedure, and remained unaware of their group allocation until the debriefing. Balanced randomization by a computer-generated algorithm in *Unipark* (Questback) ensured the concealment of the involved researchers. Participants were randomly assigned to one of four experimental study arms. They received different text-based educational material, depending on their allocation either to the active control group (CG; information only) or to one of the three narrative intervention groups (IGs; information plus three different testimonials per IG). As shown in the text boxes S1-S6 in the S1 File, the first part of the intervention consisted of general information on eMHSs, which was shown to all randomized participants. The active control group received no further information. In the second intervention part with further narrative messages, each of the three IGs were additionally exposed to three different testimonials per IG (i.e., unspecified target group, targeted at employees or at students).

At post-intervention, participants were asked to rate source credibility of information (all study arms) and of testimonials (IGs only). Next, the IGs were asked to indicate perceived similarity with testimonial sources. Then, participants filled out questionnaires on attitudes towards eMHSs and online therapies (as well as intentions to use eMHSs; reported elsewhere [45]). Finally, participants were fully debriefed (e.g., rationale for the construction of testimonials) and could indicate their current interest in registering for existing eMHSs (reported elsewhere, [45]). Upon survey completion, participants were asked to confirm the seriousness of their participation and the consent to use their data for research purposes. Completion time was 10 to 15 minutes on average.

### Stimulus material and informational interventions

The information material was adapted based on prior work [35] as well as the research literature on narrative health messages. Particularly, we refer to an equivalent rationale of the testimonials as Healey et al. [36], which is based on the taxonomy by Shaffer and Zikmund-Fisher [47]: the purpose of testimonials was persuasion (i.e., attitude change), the content focused on outcomes in terms of personal experiences with an eMHS, and the evaluative valence was positive (i.e., emphasis on helpfulness). Concerning the design of information materials, a working group of six postgraduates (M.Sc. psychology students) pre-tested all materials for comprehensibility and face validity under supervision of the principal investigator.

**Fig 2:**
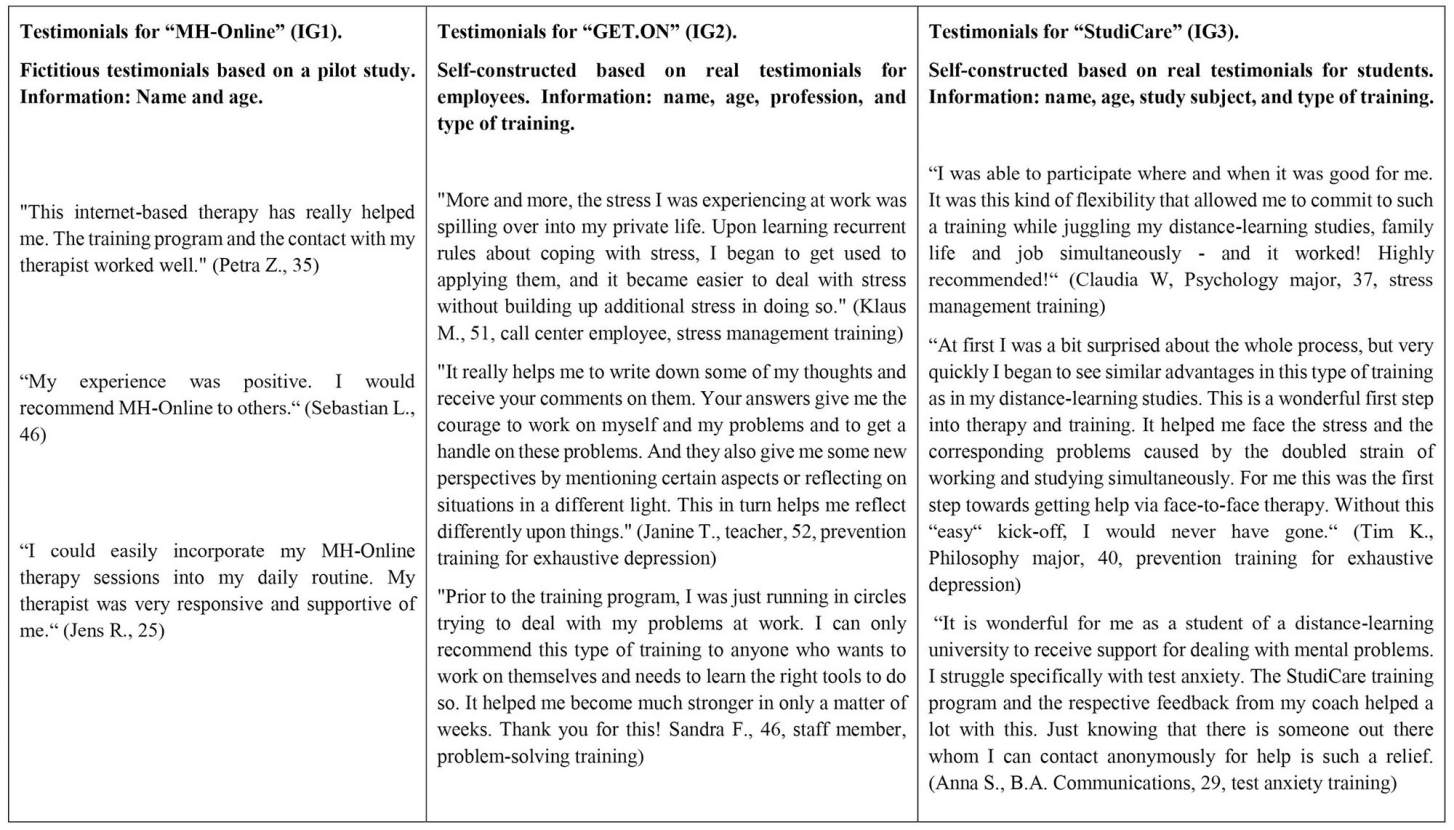
Testimonials in the narrative intervention groups in direct comparison. The texts of the stimulus materials were translated from German to English for publication purposes.

In order to investigate additional effects of targeted testimonials compared to mere information regarding attitude change, we constructed four study arms: study arm 1: CG (information only) < study arm 2: IG1 (information plus unspecific testimonials) < study arm 3: IG2 (information plus testimonials targeted at employees) < study arm 4: IG3 (information plus testimonials targeted at students). The four study arms received the same general information that also served as an active control condition (i.e., study arm 1), as shown in the Appendix S1A in S1 File. The three IGs (study arms 2–4) received additional information on eMHSs. As illustrated in Fig 2, we varied the background of testimonial sources across the three IGs in orientation to real-world samples. In the nine testimonials, we presented five female and four male personas (age range: 25–52 years). In the introductory text, the three eMHS programs, one hypothetical (“MH-Online”) and two existing (*GET*.*ON* and *StudiCare*), described to the IGs were presented with universities as providers. The central difference between the three IGs concerns the testimonials’ degree of targeting to students in terms of perceived similarity with testimonial sources.

The three testimonials for the hypothetical eMHS “MH-Online” (IG1) were modified based on the aforementioned pilot trial [35] and were considerably less detailed and less specific compared to the IGs with testimonials targeted to employees (IG2) and students (IG3), meaning that IG1 testimonials included neither information on the eMHS nor on sources’ occupation.

The two other IGs involving testimonials targeted at employees or students were designed with resemblance to real testimonials from the German websites of existing evidence-based programs on *GET*.*ON* (IG2) and *StudiCare* (IG3) that were available in 2018. We modified statements to protect personal rights and to facilitate comparability with respect to statement length and details of descriptions. Participants in IG2 received three testimonials of *GET*.*ON* offered by employees (e.g., a teacher), while IG3 read three testimonials on *StudiCare* by different sources varying in the study subject (e.g., B.A. Communications). Two of the three testimonials for the targeted information groups IG2 and IG3 described experience with identical programs (i.e., stress management [24,48,49], and exhaustive depression training [50]), while one mentioned program was target-group specific (i.e., *StudiCare* test anxiety for students in IG3 versus *GET*.*ON*’s problem solving training for employees in IG2 [51]). Due to the recruitment setting we focused on challenges and experiences presented by distance-learning students in IG3 that are also applicable to traditional students. Background information and specific needs of student personas were grounded on survey data (e.g., [9]; n = 5721 distance-learning students) and feedback by the six postgraduate (M.Sc. Psychology) students that were involved in the co-design of the stimulus material.

### Outcomes

#### Attitudes towards eMHSs for stress coping

For the pre- and post-assessment of changes in attitudes towards eMHSs for stress coping (perceived usefulness), we used five items we constructed based on theoretical considerations (e.g., Theory of Planned Behavior; TPB [52,53], Technology Acceptance Model; TAM [54]) and prior research [16,38,55]. Attitudes were assessed on a 7-point Likert scale ranging from 1 “*totally disagree*” to 7 “*totally agree*”. Baseline attitude was measured with three items, post-intervention attitude with two items, as shown in Appendix S1B in S1 File. The items for IGs were tailored by adding the name of the presented eMHS as an example. According to the conventions for social sciences [56], Cronbach’s alpha (α) in the present study was classified as good at baseline (α = .87) and mostly good at the post-intervention assessment (CG: α = .83, IG1: α = .77, IG2: α = 79, IG3: α = .86).

#### Attitudes towards online therapies

We assessed attitudes towards online therapies for common mental health problems at post-intervention using the *Attitudes towards Psychological Online Interventions* questionnaire (APOI, [55]) with 16 items and the *E-Therapy Attitudes Measure* (ETAM, [16]) with 17 items, each using a 5-point Likert scale ranging from 1 “*totally disagree*” to 5 “*totally agree*”. Both measures were tested with German-speaking populations, including clinical populations (*APOI*) and community samples (*ETAM*). We calculated overall scores as suggested [16,55]. For the *APOI*, we had to invert the items on the two scales measuring negative attitudes (i.e., skepticism and perception of risks and technologization threat) in order to build an overall score with the two positively scored subscales (i.e., confidence in the effectiveness and anonymity benefits). The *ETAM* has two components according to factor analyses (perceived usefulness/helpfulness and comparability/relative advantage) [16,35]. Higher scores in both measures indicate a more positive attitude. Despite their different scope and application fields, the correlation between the mean scores of both attitude measures was high, with a Pearson’s coefficient of *r* = .78. At post-assessment, the attitude short scale also significantly correlated with both attitude full scales (*APOI* with *r* = .66 and with *ETAM* with *r* = .58; *p*_s_<0.001). Both the *APOI* (α = .83) and the *ETAM* (α = .87) had good internal consistency scores in our study.

#### Stress perceptions

Perceived stress was measured using the validated German 10-item version of Cohen’s *Perceived Stress Scale* (PSS-10; [57]) on a 5-point Likert scale ranging from 1 “*never*” to 5 “*very often*”. We calculated mean and sum scores. The total score reflects the frequency of stressful events in the past two weeks. Cronbach’s alpha was good in our study (α = .89).

#### Source credibility

Source credibility was evaluated at post-intervention with four items we created based on the literature (e.g., [58–60]), as shown in Appendix S1B in S1 File. Two items regarding the information credibility were shown to all four study arms, while the IGs received two additional items regarding the source credibility of the presented testimonials. Each of the statements was rated on a 7-point response scale ranging from 1 “*totally disagree*” to 7 “*totally agree*”. Cronbach’s alpha was good across the four study arms (CG: α = .80, IG1: α = .86, IG2: α = 79, IG3: α = .78).

#### Perceived similarity

Perceived similarity with persons in the testimonials was assessed at post-intervention in the three IGs using five items on a scale ranging from 1 “*totally disagree*” to 7 “*totally agree*”. We constructed these items that were analyzed as mean score based on prior work (e.g., [61–63]), as presented in Appendix S1B in S1 File. Cronbach’s alpha was good in this study (IG1: α = .86, IG2: α = .89, IG3: α = .88).

#### Demographic questions and experience with eMHSs

Background variables at baseline included age in years (metric), gender (female, male, diverse), educational attainment (e.g., university entrance certification), study time model (enrolled in part- or full-time) and type of study program (traditional or distance-learning university, combination of both models, postgraduate program). As shown in Appendix S1B in S1 File, the awareness of eMHSs was assessed with the filter question (1.) “*Had you heard about e-mental health services prior to this study*?” (yes, no, not sure). If participants indicated “yes” or “not sure”, they were asked about eMHS-related experiences: (1–1.) *“Have you ever gathered information about one or more e-mental health services*?” and (1–2.) “*Have you ever used one or more e-mental health services*?” (yes, no, not sure).

### Data analyses

Only completed surveys (i.e., screened out automatically due to meeting exclusion criteria or completed per protocol) were extracted from *Unipark* (Questback) and analyzed using SPSS, version 25.0 (IBM Analytics). We excluded data sets in subsequent data cleaning for different reasons, such as being screened out (no randomization), withdrawal of consent or statement of unserious participation (check items at the end of the online survey). Other reasons were unrealistic duration of participation (e.g., much too short in terms of less than *Mean*-1 SD; i.e., 13.7–8.6 minutes = 5.1 minutes) and more than one missing value in at least one short scale (e.g., attitude short scale) or more than one missing value in full scales (*PSS-10*, *APOI*, and *ETAM*). We did not exclude outliers in subjective scales unless data suggested implausible or extreme response patterns. In other cases, we performed arithmetic mean imputation of missing values. We conducted an Intention-to-Treat (ITT) analysis. Due to the cross-sectional trial design, the nature of informed consent (no extraction of data sets of respondents who dropped out to cancel participation) and specific conditions in connection with using the virtual lab for anonymously conducted online surveys (issue of unserious participation), we included non-adherent participants, especially those with too short completion time, in the ITT analysis. Following a pre-specified interim analysis of n = 281 completed surveys in January 2019 (with n = 205 valid data sets after data cleaning; i.e., 72.9%), we recruited about one-third more participants than indicated in the power analysis (n = 436) within six months (stopping guideline). In the preliminary analyses, we tested the assumptions of different parametric tests, such as normal distribution, collinearity, variance and covariance homogeneity.

The primary outcome in terms of change in attitudes towards eMHSs for stress coping (i.e., interaction, within-subject and between-group effects) was analyzed using two-way ANOVA with repeated measures (baseline and post-assessment of the attitude short scales (H1)). To assess group differences between the four study arms (experimental variation of information) in the analyses of secondary outcomes (H2), one-way ANOVA was conducted with attitudes towards eMHSs for stress coping using the short scale on perceived usefulness (H2a) and attitudes towards online therapies (H2b) using the *ETAM* [16] and the *APOI* [55] as dependent variables. As supplementary analyses for the linear regression analysis, we also performed one-way ANOVA on information group differences regarding perceived similarity, source credibility, and perceived stress (*PSS-10* [57]) as dependent variables. In case of significant mean differences (mean_diff_), we performed post hoc tests using Tukey-HSD-adjustment if variances were equal, as indicated by Levene’s test. In case of unequal variances, Games-Howell adjustment was applied. We also calculated the Pearson’s correlation coefficient for the three attitude measures at post-intervention.

Multiple linear hierarchical regression analysis (H3) was conducted with attitude towards stress coping at post-intervention as independent variable to determine the relative influence of testimonial type (dummy-coded), perceived stress, perceived similarity and source credibility in a first step, and to control for the influence of baseline attitudes in a second step. Mediation analyses (H4) were performed using the *PROCESS* macro for *SPSS* by Hayes, version 3.4 [64], with a subsample (IG2—*GET*.*ON* vs. IG3 –*StudiCare*) and narrative information type with respect to targeting at employees versus students (dummy coding; IG2—*GET*.*ON* = 0 vs. IG3 –*StudiCare* = 1) as dichotomous independent variable (Y), perceived similarity as mediator (M) and attitudes as dependent variable (X). We tested the significance of the indirect effect using bootstrapping procedures, with 5000 samples (95% confidence interval (95% CI); model 4 in PROCESS [64]). We performed the mediation analysis with attitudes towards stress coping (H4a, attitude short scale) and online therapies (H4b, operationalized with the APOI and ETAM) as independent variables.

We classified effect sizes (ES) like partial eta-squared (*ŋ*_*p*_^2^; small ES = .01, medium ES = .06, large ES = .14), Cohen’s *d* (small ES = 0.20, medium ES = 0.50, large ES = 0.80) and *R*^2^ (small ES = .02, medium ES = .13, large ES = .26) based on Cohen’s criteria [56]. For reporting purposes, we transformed values of the effect size *ŋ*_p_^2^ to Cohen’s *d* using an online tool (*psychometrica* [65]). Data analyses for testing hypotheses were performed at an alpha level of.05 (two-tailed).

## Results

### Sample characteristics

**Fig 3:**
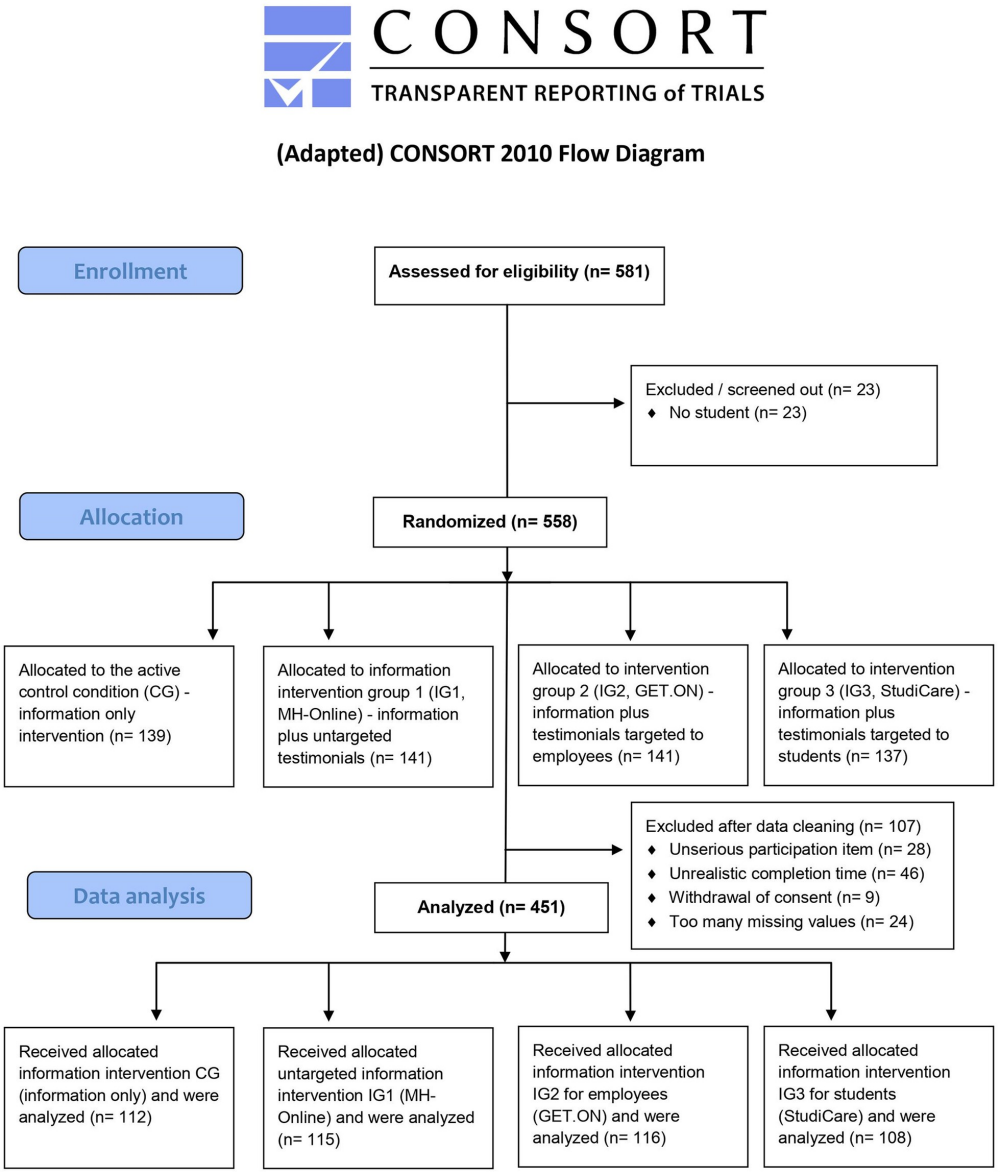
Participant flow chart. The participant flow chart for this psychological online experiment was adapted from CONSORT 2010 in conjunction with the recommendations of CONSORT-PSI 2018.

Out of N = 581 extracted data sets, we excluded n = 130 for several pre-defined reasons. As indicated in Fig 3, the final sample size was n = 451. The number of exclusions (22% of N = 581) was slightly lower than in the pre-specified interim analysis (27% of N = 281). Regarding the ITT analysis (n = 482), most data sets were excluded due to lacking consent and too many missing values in scales to generate total scores. Only n = 11 with stated unserious participation provided informed consent, but we excluded them as invalid data (i.e., control variable of the virtual lab).

Table 1 shows the demographic and educational background of our sample (n = 451), while information on the ITT sample (n = 482) is presented in S1 Table in (S2 File).

**Table 1:** Sample characteristics.

Variables		N = 451
**Gender**	Female, n (%)	340 (75.4)
	Male, n (%)	110 (24.4)
	Other/diverse, n (%)	1 (0.2)
**Age (years)**	Mean (SD),median, range (years)	32.6 (10.29),29.00, 18–65 years
**Educational attainment**^a^	General certificate of secondary school, n (%)	13 (2.9)
	Master craftsman’s diploma, n (%)	10 (2.2)
	Advanced college entrance qualification, n (%)	28 (6.2)
	University entrance qualification, n (%)	188 (41.7)
	College or university degree, n (%)	173 (38.3)
	• Bachelor’s degree, n (%)	94 (20.8)
	• Master’s degree, n (%)	57 (12.6)
	• PhD level, n (%)	22 (4.9)
	Other, n (%)	39 (8.6)
**Study program**	Distance-learning university, n (%)	400 (88.7)
	Traditional (on-campus) college or university, n (%)	27 (6.0)
	Distance-learning and traditional study program, n (%)	23 (5.1)
	Postgraduate program, n (%)	1 (0.2)
**Study time model**	Studying in full-time, n (%)	233 (51.7)
	Studying in part-time, n (%)	217 (48.1)
	Missing (postgraduate program), n (%)	1 (0.2)
**Awareness of e-mental health services**^b^	No	224 (49.7)
	Yes	191 (42.4)
	Not sure	36 (8.0)

N = 451.

^a^Educational attainment refers to the German education system.

^b^Question: “Had you heard about e-mental health services prior to this study?”.

As shown in Table 1, n = 227 students (50.3%) reported to have been either aware of the existence of eMHSs (n = 191) prior to this study or to be not sure (n = 36). In this segment, n = 75 students (33.0% of n = 227) stated to have purposefully sought information on one or more eMHSs in the past, while most (n = 142, 62.6%) had not (not sure: n = 10/227; 4.4%). The vast majority (n = 183/227, 80.6%) indicated to have never used any eMHS. The remaining n = 32 students (14.1% of n = 227; 7.1% of N = 451, respectively) indicated to have already used one or more eMHSs, whereas n = 12 (5.3% of n = 227; 2.7% of N = 451, respectively) were not sure.

As shown in S2 File, the stress level according to the PSS-10 was moderately high (*Mean* = 27.04, *SD* = 6.64, adapted scale range: 1–5; original scale range: 0–4).

### Main analyses

#### Primary outcome

*H1*: *Effects of testimonials on attitudes toward eMHSs for stress coping*. Attitudes were already positive at baseline (*M* = 5.10, *SD* = 1.08; range: 1–7). No significant positive attitude change was identified within or between the four experimental groups. Two-way ANOVA on the short scale on attitudes towards eMHSs for stress coping (perceived usefulness) yielded neither a significant interaction between time x group, *F*_(3, 447)_ = 1.48, *p* = 0.220, *ŋ*_*p*_^2^ = .01 (*d* = 0.20), nor a main effect of time, *F*_(1, 447)_ = 1.02, *p* = 0.313, *ŋ*_*p*_^2^ = .002 (*d* = 0.09) or group, *F*_(3, 447)_ = 1.58, *p* = 0.193, *ŋ*_*p*_^2^ = .01 (*d* = 0.20). The rejection of H1 was confirmed by the ITT analysis (n = 482), with no time x group interaction, *F*_(3, 478)_ = 1.61, *p* = 0.186, *ŋ*_*p*_^2^ = .01 (*d* = 0.20), and no main effect of time, *F*_(1, 478)_ = 0.86, *p* = 0.354, *ŋ*_*p*_^2^ = .002 (*d* = 0.09), and group, *F*_(3, 478)_ = 1.33, *p* = 0.263, *ŋ*_*p*_^2^ = .008 (*d* = 0.18).

#### Secondary outcomes

*H2*: *Information type group differences at post-intervention*. Table 2 shows descriptive data on the attitude scales, all with moderately high scores on average (i.e., rather positive attitudes towards eMHSs for stress coping and therapy). At baseline, there was a marginally significant difference in one-way ANOVA regarding attitudes towards eMHSs for stress coping, with a small ES (*F*_(3,447)_ = 2.67, *p* = 0.047, *ŋ*_*p*_^2^ = .02; *d* = 0.29). Tuckey-HSD adjusted post hoc tests showed that participants assigned to IG2 (testimonials for employees) reported more positive attitudes than those in IG1 (unspecific testimonials) at baseline (mean_diff_ = 0.39, *SE* = 0.14, *p* = 0.033, 95% CI [0.02, 0.75]; *d* = 0.37). The Kruskal-Wallis H-Test (one-way ANOVA by ranks) did not indicate such differences between the groups at baseline (*p* = 0.079). In the ITT analysis (n = 482), there was also no significant between-group difference regarding attitudes towards eMHSs for stress coping at baseline, as indicated by the one-way ANOVA (*F*_(3,478)_ = 2.44, *p* = 0.064, *ŋ*_*p*_^2^ = .015; *d* = 0.25) and the H-Test (*p* = 0.069).

**Table 2:** Descriptive data and between-group differences in attitudes towards e-mental health services and online therapies among university students.

Study arm/metrics	Range scale	Overall (n = 451)	CG (n = 112)	IG 1: MH-Online(n = 115)	IG 2: GET.ON (n = 116)	IG 3:StudiCare (n = 108)	Difference^a^^,^^b^, effect size	Post hoc tests^c^^,^^d^^,^^e^
Measure/construct	range	M (SD)	M (SD)	M (SD)	M (SD)	M (SD)	F-Test	adjusted
Attitude towards eMHSs for stress coping (Baseline), 3 items	1–7 (1.33–7.00)	5.10 (1.08)	5.04 (1.16)	4.93 (1.11)	5.32 (0.99)	5.09 (1.02)	*F*_(3,447)_ = 2.67, *p* = 0.047, ŋ_p_^2^ = .02^a^ in H-Test, n.s (*p* = 0.079)^b^	More positive in IG2 than in IG1^c^ (*p* = 0.033), H-Test, N.A.^b^^,^^e^
Attitude towards eMHSs for stress coping (Post), 2 items	1–7 (1.00–7.00)	5.14 (1.13)	5.06 (1.27)	5.08 (1.17)	5.24 (1.01)	5.16 (1.08)	*F*_(3,447)_ = 0.58, *p* = 0.628, ŋ_p_^2^ = .004	N.A. (n.s.)^e^
Attitude towards online interventions (APOI—Post only), 16 items^f^	1–5 (1.50–4.75)	3.13 (0.52)	3.03 (0.58)	3.07 (0.54)	3.21 (0.45)	3.21 (0.50)	*F*_(3,447)_ = 3.46, *p* = 0.016, ŋ_p_^2^ = .02	More positive in IG2 and IG3 than in IG1 and the CG^d^ (*p*_*s*_<0.05)
Attitude—online therapy (ETAM—Post only), 17 items^f^	1–5 (1.35–4.71)	3.08 (0.56)	3.03 (0.62)	3.02 (0.57)	3.16 (0.51)	3.10 (0.50)	*F*_(3,447)_ = 1.57, *p* = 0.197, ŋ_p_^2^ = .01	N.A. (n.s.)^e^

N = 451; CG = control group (“information only”), IG = intervention group; IG1 = MH-Online (hypothetical program, testimonials by user from an unspecified target group); IG2 = GET.ON (testimonials by employees); IG3 = StudiCare (testimonials by university students); eMHSs = electronic mental health services, Post = Post-intervention assessment; APOI = attitudes towards psychological online interventions (questionnaire), ETAM = e-therapy measure (questionnaire).

^a^One-way ANOVA.

^b^Kruskal-Wallis H-test (conducted due to the uncertain multivariate normal distribution of the attitude short scale).

^c^Post hoc test with Tuckey-HSD adjustment.

^d^Post hoc test with LSD-adjustment (least significant difference test).

^e^N.A. = not applicable for post hoc tests (n.s. = not significant).

^f^The APOI and ETAM with 33 items were only measured at post-intervention due to the scope and length of the survey.

*H2a*: *Attitudes towards eMHSs for stress coping*. We found no evidence for more positive attitudes towards eMHSs for stress coping in IG3 after exposure to testimonials targeted at students (*p* = 0.628). In view of the insignificant findings in the two-way ANOVA (H1), we report the results of the one-way ANOVA for the attitude short scale (pre- and post-assessment) only for the sake of completeness in Table 2. Accordingly, comparisons between effect sizes of outcome measures regarding attitudes towards eMHSs for stress coping (short scale) versus online therapies (*ETAM* and *APOI*) at post-intervention were not performed (H2-sub-hypotheses, research question). Effect sizes were overall small, as demonstrated in Table 2.

*H2b*: *Attitudes towards online therapies*. At post-intervention, one-way ANOVA demonstrated no differences in attitudes towards online therapies according to the *ETAM* between the four study arms (*p* = 0.197), as shown in Table 2.

Only in case of an operationalization of attitudes towards online therapies using the *APOI*, a significant group difference with a small ES was found by one-way ANOVA, *F*_(3,447)_ = 3.46, *p* = 0.016, *ŋ*_*p*_^2^ = .02 (*d* = 0.29). Tukey-HSD adjusted post hoc tests showed no significant differences in attitude (*p*_*s*_>0.05). Only liberal LSD-adjusted post hoc tests indicated significantly higher *APOI* scores of participants in IG2 (employee’s testimonials on *GET*.*ON*) than in the CG (“information only”; mean_diff_ = 0.17, *SE* = 0.07; *p* = 0.012, 95% CI ⦋0.04, 0.31⦌; *d* = 0.35) and in IG1 (unspecific testimonials on the hypothetical “MH-Online”; mean_diff_ = 0.14, *SE* = 0.07; *p* = 0.044, 95% CI ⦋0.004, 0.27⦌; *d* = 0.28), all with small ES. There was no difference in attitudes, as measured via the *APOI*, between IG2 after exposure to testimonials targeted at employees (*GET*.*ON*) and IG3 that received testimonials targeted at students (*StudiCare*; mean_diff_ = 0.001, *SE* = 0.07; *p* = 0.994, 95% CI ⦋-0.14, 0.14⦌; *d* = 0.0). Consequently, like in IG2, student-targeted information in IG3 (*StudiCare*) was associated with significantly more positive attitudes than in the CG (mean_diff_ = 0.17, *SE* = 0.07; *p* = 0.014, 95% CI ⦋0.04, 0.31⦌; *d* = 0.33), while the difference to IG1 was marginally significant (“MH-Online”; mean_diff_ = 0.14, *SE* = 0.07; *p* = 0.049, 95% CI ⦋0.001, 0.27⦌; *d* = 0.27).

The ITT analysis (n = 482) confirmed the null findings for the attitude short scale on stress coping (H2a) and the *ETAM* (H2b), and the significant small effect on attitudes according to the *APOI* (H2b), which was in favor of targeted testimonials, *F*_(3, 478)_ = 3.81, *p* = 0.010, ŋp^2^ = .02 (*d* = 0.31).

*H3*: *Determinants of attitudes toward eMHSs for stress coping*. As shown in Table 3, in step 2 of the hierarchical regression model perceived similarity (*ß* = .10) and source credibility (*ß* = .30) still had a significant positive but decreased influence on attitudes towards eMHSs for stress coping at post-intervention assessment after controlling for the large influence of baseline attitude (*ß* = .58, *p*_*s*_<0.001). There was neither a statistically significant influence of perceived stress nor of narrative information type in both steps (IG3vs.IG2 and IG3vs.IG1; *p*_s_>0.05). S2 Table in S2 File shows differences in the determinants between the study arms. For instance, the three IGs (n = 339) differed significantly regarding perceived similarity (*ŋ*_*p*_^2^ = .15, *d* = 0.84), with highest scores in IG3 presenting testimonials targeted at students.

**Table 3:** Hierarchical multiple regression of information type, perceived stress, perceived similarity and source credibility on attitudes towards e-mental health services for stress coping.

**Step 1**	**Variable**^a^	**B**	***SE* (B)**	**ß**	**T**	***p*-value**	**95% CI (B) 1.53**	
	(Constant)	.86	0.34		2.53	0.012	0.19	1.53
	IG3 vs. IG2^b^	.24	0.14	.09	1.72	0.086	-0.03	0.51
	IG3 vs. IG1^c^	.23	0.13	.09	1.79	0.074	-0.02	0.49
	Stress Level	.09	0.07	.05	1.32	0.188	-0.05	0.23
	Perceived Similarity	.23	0.05	.20	4.48	<0.001	0.13	0.34
*R*^2^ = .32^d^	Source Credibility	.58	0.05	.52	12.98	<0.001	0.49	0.67
**Step 2**	**Variable**	**B**	***SE* (B)**	**ß**	**T**	***p*-value**	**95% CI (B) 1.53 Step**	
	(Constant)	-.41	0.27		-1.51	0.133	-0.95	0.13
	IG3 vs. IG2^b^	.01	0.11	.004	0.10	0.920	-0.20	0.22
	IG3 vs. IG1^c^	.19	0.10	.07	1.84	0.066	-0.01	0.38
	Stress Level	.08	0.05	.05	1.43	0.155	-0.03	0.18
	Perceived Similarity	.12	0.04	.10	2.85	0.005	0.04	0.20
	Source Credibility	.33	0.04	.30	8.91	<0.001	0.26	0.41
*R*^2^ = .60^e^	*Baseline Attitude*	.61	0.04	.58	17.41	<0.001	0.55	0.68

N = 451. Dependent variable: Attitude towards eMHSs for stress coping at post-intervention (perceived usefulness); IG1 = testimonials for MH-Online (unspecific audience), IG2 = testimonials for GET.ON (employees), IG3 = testimonials for StudiCare (students), *SE* = standard error B, CI = confidence interval. B = unstandardized coefficient; β = standardized coefficient (beta-weight).

^a^Values of the CG for items on perceived similarity and credibility of testimonials were imputed (mean imputation).

^b^Information type, dummy coding: IG3 vs. IG2 = “Dummy_IG2vsIG3”(IG1 = 0, IG2 = 1, IG3 = 0).

^c^Information type, dummy coding: IG3 vs. IG1 = “Dummy_IG1vsIG3”(IG1 = 1, IG2 = 0, IG3 = 0).

^d^Step 1: *R*^2^ = .32, *F*_(5,445)_ = 42.14, *p*<0.001 (adjusted *R*^2^ = .31).

^e^Step 2: *R*^2^ = .60, *F*_(1,444)_ = 303.25, *p*<0.001 (adjusted *R*^2^ = .59), *R*^*2*^ increase: Δ*R*^*2*^ = .28.

In the ITT-analysis (n = 482), perceived similarity (*ß* = .10, *p* = 0.003) and source credibility (*ß* = .31, *p*<0.001) also had a positive influence on attitudes towards eMHSs for stress coping in step 2 of the hierarchical regression model, after controlling for baseline attitudes (*ß* = 58, *p*<0.001). In contrast to the per protocol analysis there was also a statistically significant albeit small influence of targeted vs. untargeted information type (IG3 vs. IG1; *ß* = .07, *p* = 0.039), as shown in S3 Table in the S2 File.

*H4*: *Mediating effects of perceived similarity in targeted testimonials on attitude*. *H4a*: *Mediating effects regarding attitudes towards eMHSs for stress coping*. Mediation analyses were performed with the subsample of 224 participants assigned to one of two groups receiving targeted testimonials for employees or students (IG2 = *GET*.*ON* vs. IG3 = *StudiCare*) and with perceived similarity as mediator, since both targeted IGs did not differ in source credibility ratings (see, S2 File) and were, unlike IG1, dealing with existing eMHSs.

**Fig 4:**
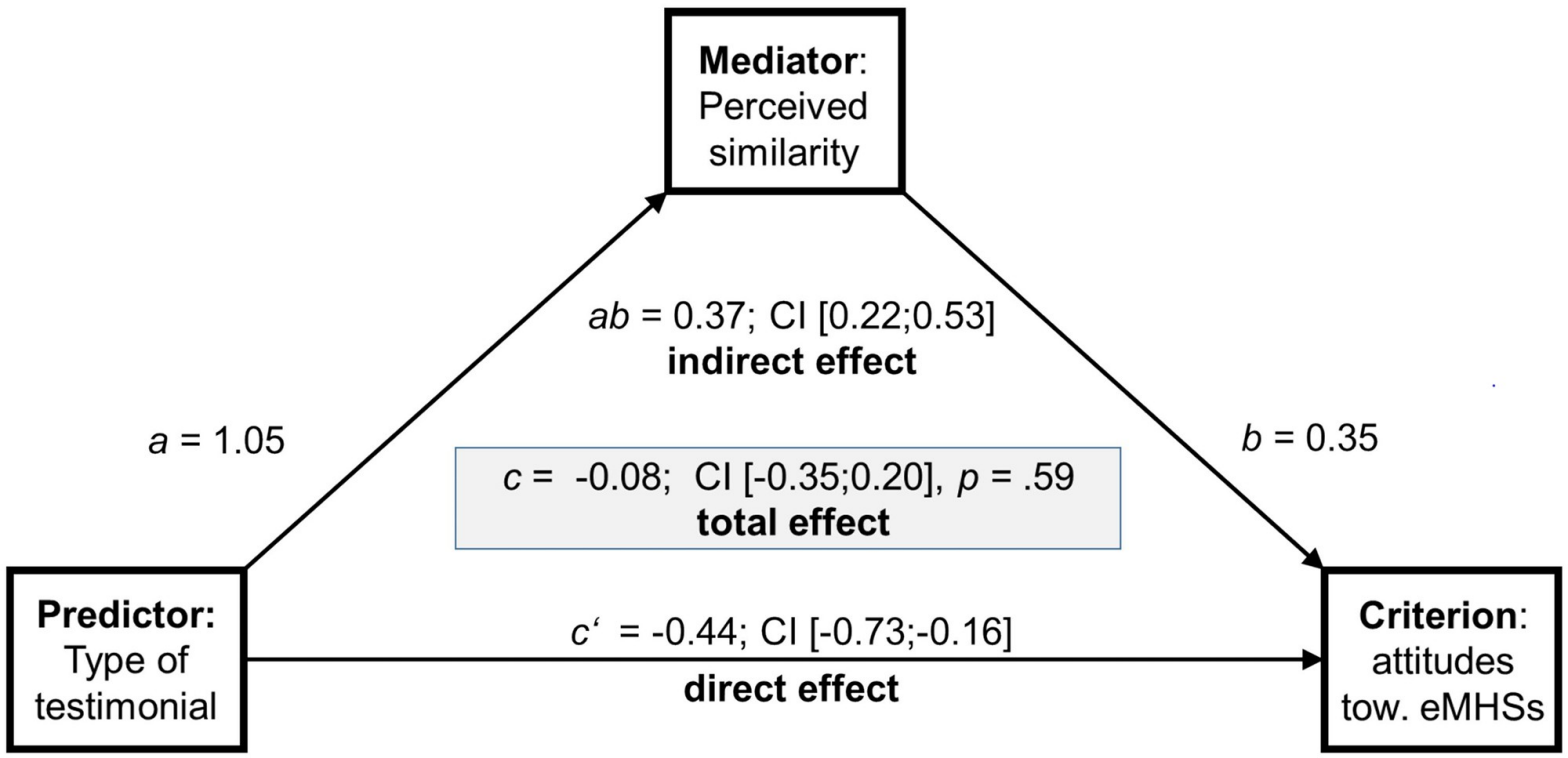
Mediation effect of perceived similarity in the relationship between targeted testimonials and attitudes towards e-mental health services for stress coping. Partial mediation effect of perceived similarity in the relationship between the type of targeted testimonials (students versus employees) and attitude towards eMHSs for stress coping (n = 224). CI = 95% confidence interval (95% CI). The effect is defined as significant if the confidence interval does not include the number zero; 5000 bootstrapping samples.

As illustrated in Fig 4, there was a significant direct effect of target-group specific testimonials (students versus employees) on attitude towards eMHSs for stress coping (c’-path = -0.44, 95% CI [-0.73, -0.16]) and a significant indirect effect of perceived similarity in the relationship between targeted testimonial source and attitude (ab-path = 0.35, partially standardized indirect effect; 95% CI [0.22, 0.53]). The insignificant total effect (c-path = -0.08; 95% CI [-0.35, 0.20]) suggests suppressing effects in terms of pathways with opposite signs (dummy coding, IG2 vs. IG3). This means that there was a partial mediating effect of perceived similarity in the relationship between testimonial type and attitude in favour of student-targeted testimonials on *StudiCare*.

The ITT analysis (n = 241) confirmed the partial mediating effect of perceived similarity on attitudes towards eMHSs for stress coping from the per protocol analysis, including a significant direct effect (c’-path = -0.48, 95% CI [-0.77, -0.20]), a significant indirect effect of perceived similarity on attitude (partially standardized indirect effect; ab-path = 0.33, 95% CI [0.21, 0.48]) and an insignificant total effect (c-path = -0.11; 95% CI [-0.40, 0.17]).

*H4b*: *Mediating effects regarding attitudes towards online therapies*. As shown in the S2 File, there was also a partial mediation of perceived similarity regarding attitudes toward online therapies (n = 224, per protocol) measured via the *ETAM*, including a significant direct effect of targeted testimonials (c’-path = -0.15, 95% CI [-0.30, -0.01]) and an indirect effect of perceived similarity on attitudes (ab-path = 0.19, partially standardized indirect effect; 95% CI [0.07, 0.32]) as well as an insignificant total effect (c-path = -0.06; 95% CI [-0.19, 0.08]). Additionally, perceived similarity fully mediated the association between targeted testimonials and attitudes toward online interventions in the per protocol analysis (n = 224), if measured using the *APOI* (ab-path = 0.22, partially standardized indirect effect, 95% CI [0.09, 0.36]).

As demonstrated in the S2 File, the ITT analyses (n = 241) again confirmed the partial mediation for the *ETAM* (ab-path = 0.21, partially standardized indirect effect, 95% CI [0.09, 0.33]) and a full mediation effect for the *APOI* (ab-path = 0.21, partially standardized indirect effect, 95% CI [0.10, 0.32]).

## Discussion

The purpose of this online experiment was to explore the effects of information on attitudes towards eMHSs among university students. While several assumptions on testimonial effects were not confirmed, the main contribution of this study can be nonetheless seen in the transfer of findings on the role of perceived similarity in narratives messages from other contexts to eMHSs. Another general contribution concerns the critical, context-sensitive appraisal on whether user testimonials add any value regarding promoting the acceptance of eMHSs among university students.

*H1*: *Information effects on attitude change*. In light of previous research and theoretical considerations, we assumed that providing information on eMHSs has the potential to improve attitudes among university students. Yet, we found no significant momentary change in attitudes towards eMHSs for stress coping. The most obvious explanation is that attitudes in our study were already relatively positive at the baseline measurement so that it would have been difficult to achieve further improvements with this simple narrative intervention. Potentially, the recruitment of students who are usually digital natives, and especially psychology students, may have contributed to ceiling effects. Interestingly, only less than half of the sample (42%) reported to have been aware of the existence of eMHSs, and very few (7%) indicated any prior experience with such offers. Previous research has shown that undergraduates without personal experience with treatments for mental health problems are more likely to be influenced by the testimonials of previous users than those with personal experience [25]. In addition, this finding partly contradicts prior studies indicating an association between little experience and less favorable views on eMHSs in particular [15,20,66]. Possibly, positive attitudes are not directly transferable to user behavior or experience and can thus not automatically be treated as key precondition for the adoption of eMHSs by university students, as earlier research suggested [19]. Another point to consider in this context is that the recruitment material for this study included a positively framed definition of eMHSs, which potentially may have unintendedly shaped expectations amongst later participants and, by these means, intensified the self-selection of students being interested in digital health. In turn, however, it can be stated that the moderately high stress levels in this study suggest that students with a need for stress management interventions decided to take part in the study and thereby addressed a target group of interest.

*H2*: *Group differences regarding the effects of narrative information*. Furthermore, our results indicated limited evidence for an impact of target-group specific testimonials on attitudes towards eMHSs. Only in case of the assessment of attitudes toward online therapies using the *APOI*, we identified a significant albeit weak positive influence in favor of targeted testimonials, which is in accordance in other research scoping on digitalized treatments [34]. In contrast, there were no group differences regarding the attitude short scale on perceived usefulness of eMHSs for stress coping and the *ETAM*. These results fit into the limited and inconsistent findings on the usefulness of testimonials in mental health promotion and the overall inconclusive evidence base in health communication, which appears to be highly context-sensitive [26]. Although these null findings for testimonials are in accordance with earlier research using similar stimulus material [35], it was unexpected to identify no meaningful effects of information and group differences at all, as we put more emphasis on source credibility and similarity and addressed specific intervention fields, such as test anxiety for students. Especially, the results on lacking testimonial effects contradict the hypothesis that little experience with specific mental health services would be a driver of the heuristic formation of attitudes in the sense of a greater impact of the opinions by past users on mental health interventions [25,34]. Using more sophisticated information approaches might better address students´ preferences and help alter attitudes. In line with this assumption, psychological resistance -as possible negative response to testimonial exposure- has been previously found among academic audiences, such as college students [67]. In particular, one-third of our participants held at least a bachelor’s degree, and we recruited psychology students being familiar with quantitative research methods. This might contribute to more skeptical views on anecdotal evidence, or in our case, help explain the ineffectiveness of testimonials. Moreover, meta-analytic evidence [29] indicated a stronger influence of statistical compared to narrative health information on attitudes and beliefs, while narrative information was found to be more effective in changing behavioral intentions. Concerning implications for the design of a future trial, it may be worthwhile to combine narrative with statistical information [34], including references to systematic reviews on eMHSs for students (e.g., [13,14]).

*H3*: *Determinants of attitudes*. We have also investigated potential determinants of attitudes towards eMHSs for stress coping following the exposure to targeted information, and after taking the influence of baseline attitudes into account. As expected, we found statistically significant influences of source credibility and perceived similarity on attitudes towards eMHSs, both representing well-established features of health narratives [28]. Interestingly, perceived stress as a surrogate variable or proxy for personal need was not associated with attitudes towards eMHSs for stress coping. In this context, it should be noted that perceived stress in the *PSS-10* was higher in our sample than average scores reported in a community-representative sample from Germany [57]. Potentially, some participants were already motivated to seek support or had own experience with other types of mental health promotion interventions and were thus less likely to be affected by narrative information. In line with the *ELM* [30], personal involvement and interest may contribute to systematic information processing and may reduce the impact of testimonials based on heuristics. Accordingly, earlier research indicated that the perceptions of testimonials by unconcerned people may differ from those with past or current health problems on the one hand [33]. Yet, some studies demonstrated positive effects of testimonials on hypothetical psychological treatment decisions [25,34]. The unexpected high stress levels for a nonclinical sample corresponds to international surveys showing that stress-associated mental health problems are more prevalent among university students [1–3]. Beyond providing information on psychological services, it could therefore be helpful to generally educate students and university staff about stress and indicated mental health prevention programs [68].

*H4*: *Mediating effects of perceived similarity*. Finally, we assessed perceived similarity as an underlying mechanism of testimonial effects. In line with our assumptions, we demonstrated a partially mediating effect of perceived similarity on attitudes towards eMHSs for stress coping and online therapies according to the *ETAM* after exposure to targeted testimonials, as well as a full mediation effect for the attitude measurement using the *APOI*. This finding not only confirms perceived similarity with testimonial sources as a persuasive factor in narrative messages from various other health fields [26] but also extends it to the digital mental health context. Future research could focus on the incremental effects of individual tailoring of testimonials to study subjects (e.g., medicine) as well as aspired professions of students (e.g., physician) in more detail. Investigations of further variables such as personal involvement (e.g., individual health risk) that could influence attitudes, as outlined in the *ELM*, may be fruitful as well. Preferably, a discrete-choice conjoint experiment could be conducted to identify preferences and tradeoffs between relevant features of multi-component mental health information strategies [69].

### Limitations

This trial has several limitations that should be taken into account.

First, a major drawback of the online recruitment is related to the self-selection bias, restricting the generalizability of our findings to student samples in general. Particularly, the recruitment sources were mainly targeted at psychology students. Since we mainly recruited within one university setting, we did not ask for the study disciplines and relied on a minimal set of background questions to reduce the risk of linking data to individuals. Most participants were distance-learning students (89%), who often differ from traditional university students in several ways, such as older age and different employment status [9]. Furthermore, the gender imbalance in our study (75% women) may be a relevant limitation for assessing testimonial effects [26]. Potentially, with respect to a rather high percentage of students with academic degrees (38.3% bachelor or above) the academic background may have buffered such gender effects. Although this gender imbalance is quite usual for psychological surveys and women are more likely to access mental health services [70,71], it should be mentioned that studies like ours provide few insights into online help-seeking determinants especially of young men [72]. Nevertheless, the stress levels of participants indicate that we have reached a relevant target group with a need for support that has not been aware of eMHSs but is quite open to this intervention approach.

Second, due to connection with a virtual lab we expected a high response rate of distance-learning students. We therefore chose characteristics of the testimonial sources reflecting their larger demographic diversity and their views. Hence, the highest match regarding perceived similarity with testimonial sources in IG3, with a large effect size, might be mainly attributable to the overweight of distance-learning students in our sample.

Third, it appears very likely that study credits were the major incentive for participation in this online study and probably the main reason for the exclusion of n = 130 invalid data sets due to unserious participation (i.e., 22% of the initial sample size). Unrealistic completion time (e.g., less than five minutes) was the most common reason for exclusion (n = 46). Gift cards could have increased the response rate of a broader range of students.

Fourth, we may have used too much technical language in the information material, mainly reflecting the perspectives and knowledge of postgraduate psychology students. In addition, unlike many real-world testimonials we included no photos of testimonial sources in order to keep the experimental variations manageable and limit the amount of unsystematic variation. Moreover, respondents consciously participated in an experiment on information effects, which further limits the external validity of our study.

Finally, due to a lack of suitable scales for the assessment of some constructs such as perceived similarity we needed to adapt or create items. This poses an issue for the construct validity. Nonetheless, we calculated internal reliability scores and conducted pre-tests for comprehensibility. In addition, attitudes toward online therapies were measured with established multi-item questionnaires in order to buffer methodological shortcomings of short scales [38]. In contrast to the attitude short scale assessing perceived usefulness, we did not use the two full-scale attitude measures with a total of 33 items (*APOI* with 16 items, *ETAM* with 17 items) at baseline to keep the response burden as low as possible (max. 15 minutes completion time). Therefore, the primary outcome was only assessed regarding changes in attitudes towards eMHSs for stress coping.

Taken together, our results should be seen as preliminary and need to be interpreted with caution.

### Conclusions

This study showed favorable views on eMHSs among moderately distressed university students regardless of their little experience with such offers, and that these attitudes were not affected or further improved by information involving testimonials. Hence, positive attitudes towards eMHSs may not require personal user experience and probably represent no sufficient precondition for their adoption by university students. Overall, our results indicated at least limited evidence for benefits of targeted information and testimonials on attitudes towards eMHSs. Accordingly, another main contribution of this study concerns the identified mediating effects of perceived similarity on attitudes towards eMHSs for stress coping and online therapy, which extends prior research from health communication and should be further investigated in future experiments. Principally, there seems to be no universal “one-size-fits-all” approach to inform about novel psychological options, but easily comprehensible, tailored narrative messages may provide one starting point to increase the awareness of eMHSs. Yet, it remains unclear which information strategies are most suitable for university students. In our study, participants reported moderately high stress levels on average, and may thus have potentially benefited more from detailed psychoeducational information on mental health services for stress coping. Our findings also challenge the common practice of using testimonials for the promotion and design of eMHS programs without empirical justification. It still has to be debated whether testimonials add any value to other information strategies in this context. Further research is thus needed to determine the usefulness of narrative messages and the optimal design of eMHS information.

## Supporting information

S1 File Appendix S1. Supplementary material on the methods section.
(PDF)

S2 File Appendix S2. Supplementary material on the results section.
(PDF)

S3 File Appendix S3. CONSORT-PSI 2018 checklist.
(PDF)
