# Supplementary material for: Exploring the influence of testimonial source on attitudes towards e-mental health interventions among university students: Four-group randomized controlled trial
Source: PLoS One. 2021 May 26;16(5):e0252012. doi: 10.1371/journal.pone.0252012 (PMC8153476; doi:10.1371/journal.pone.0252012)
Supplement: S1 File — Supplementary material on the methods section. (PDF) [file pone.0252012.s001.pdf]

Supporting Information

S1 File. Appendix 1 on the Methods section.

Appendix 1A: Methods Information and testimonial texts

Notes: The complete original stimulus material, the original online questionnaire and data sets of final (n=451) and intention-to-treat (n=482) analyses, can be accessed via the GESIS repository (German "Leibniz Institut für Sozialwissenschaften"; SowiDataNet – datatorium); DOI: https://doi.org/10.7802/2127) by researchers for noncommercial use.

The study was conducted in German language. All text-based materials we used in the present study were translated from German to English for publication purposes. The original questionnaire is accessible via GESIS.
Supplementary Textbox S1 illustrates general information that was shown to the four study arms. The active control condition (CG, information only) received no further information, while the three narrative information groups (IGs) were asked to read three testimonials.

General information, as shown to the four experimental groups

Supplementary Textbox S1. General information on eMHSs.

"The following text will provide you with information on guided electronic mental health trainings and therapies to help cope with psychological problems such as stress and to build resilience. Please read the description carefully and answer the questions regarding your opinion on these new interventions afterwards. There will be no questions to test your knowledge of these interventions.

Electronic mental health trainings and therapies with regards to mental health offer new opportunities to improve and/or expand care for afflicted persons. Mental illness often remains untreated. Internet-based psychotherapy can be considered a means to bridge the gap and offer care to more individuals. Numerous scientific studies have shown the efficacy of internet-based therapy for a broad range of psychological problems that can for instance arise from prolonged exposure to stress.

These internet-based interventions employ methods from Cognitive Behavioral Therapy that are also used in regular face-to-face therapy sessions. For instance, there are trainings to generally help cope with stress, to build resilience, to cope with mild or moderate depression, anxiety disorders or eating disorders. One of the currently existing forms are internet-based guided programs that allow patient to access online programs or online self-learning resources with the help of a therapist. Daily use of an app can also be employed. Patients communicate with their therapist via e-mail, chat or video conference. Flexibility regarding time and location is seen as a big advantage."

S1 File. Supporting information.

Additional information and testimonials (IGs only)

At first, participants in the three IGs read the following passage in which the name of the eMHS (X) and the provider (name of university; Y) was adapted to the IG:

> (…) "[X] ([Y]) offers evidence-based and professionally guided internet-based training programs in areas such as "stress", "resilience", and "depression/anxiety." ***
>
> *** IG 1: X="MH-Online" (Y=Albert-Ludwigs-Universität Freiburg); IG 2: X="The GET.ON Institute" (Y=Leuphana Universität Lüneburg); IG 3: X= "StudiCare" (Y=Friedrich-Alexander-Universität Erlangen)

In the condition receiving testimonials for GET.ON (IG2), we added a sentence to suggest a broader audience than for *StudiCare* (IG3):

> "Participation is open for various social groups, e.g. employed persons, trainees, students, retired persons."

Textbox S2 shows additional information on eMHSs presented to the three IGs (X = name of the eMHS program).

Supplementary Textbox S2. Introduction to eMHSs in the IGs.

"These online trainings consist of 6 consecutive one-hour sessions. You can accompany the online sessions with an app that allows for daily use. Additionally, you may receive personal coaching sessions. The training program aims to help you regain productivity. The resilience-training program includes sessions on self-efficacy, optimism, relationships, and self-care. During the stress management training programs, participants learn to identify personal issues, practice coping mechanisms, apply problem-solving techniques, and learn how to manage difficult emotions.

Individuals who have participated in [X]'s training programs were asked to relate their experiences:"

Next, three testimonials were shown per IG, as shown in the Textboxes S3 to S5.

Supplementary Textbox S3. Testimonials for “MH-Online” (IG1).

"This internet-based therapy has really helped me. The training program and the contact with my therapist worked well." (Petra Z., 35)

“My experience was positive. I would recommend MH-Online to others.“ (Sebastian L., 46)

“I could easily incorporate my MH-Online therapy sessions into my daily routine. My therapist was very responsive and supportive of me.“ (Jens R., 25)

Participants in IG 2 received three testimonials targeted to employees (i.e. *GET.ON* interventions) presented by employee personas that varied in age, gender, occupation and type of mental health training (Textbox S4).

Supplementary Textbox S4. Testimonials for “GET.ON” (IG2).

"More and more, the stress I was experiencing at work was spilling over into my private life. Upon learning recurrent rules about coping with stress, I began to get used to applying them, and it became easier to deal with stress without building up additional stress in doing so." (Klaus M., 51, call center employee, stress management training)

"Prior to the training program, I was just running in circles trying to deal with my problems at work. I can only recommend this type of training to anyone who wants to work on themselves and needs to learn the right tools to do so. It helped me become much stronger in only a matter of weeks. Thank you for this! Sandra F., 46, staff member, problem-solving training)

"It really helps me to write down some of my thoughts and receive your comments on them. Your answers give me the courage to work on myself and my problems and to get a handle on these problems. And they also give me some new perspectives by mentioning certain aspects or reflecting on situations in a different light. This in turn helps me reflect differently upon things." (Janine T., teacher, 52, prevention training for exhaustive depression)

Participants in IG 3 read three testimonials targeted to students (*StudiCare* trainings, see Textbox S5). Due to the employment of the virtual lab of the University of Hagen, we focused on experiences presented by distance-learning students that were also applicable to traditional students.

Supplementary Textbox S5. Testimonials for "StudiCare" (IG3).

"It is wonderful for me as a student of a distance-learning university to receive support for dealing with mental problems. I struggle specifically with test anxiety. The StudiCare training program and the respective feedback from my coach helped a lot with this. Just knowing that there is someone out there whom I can contact anonymously for help is such a relief. (Anna S., B.A. Communications, 29, test anxiety training)

"At first I was a bit surprised about the whole process, but very quickly I began to see similar advantages in this type of training as in my distance-learning studies. This is a wonderful first step into therapy and training. It helped me face the stress and the corresponding problems caused by the doubled strain of working and studying simultaneously. For me this was the first step towards getting help via face-to-face therapy. Without this "easy" kick-off, I would never have gone." (Tim K., Philosophy major, 40, prevention training for exhaustive depression)

"I was able to participate where and when it was good for me. It was this kind of flexibility that allowed me to commit to such a training while juggling my distance-learning studies, family life and job simultaneously - and it worked! Highly recommended!" (Claudia W, Psychology major, 37, stress management training)

Supplementary Textbox S6 shows the testimonial texts for the three IGs in direct comparison.

Supplementary Textbox S6. Contrasting the three testimonials per intervention group.

Testimonials for "MH-Online" (IG1).	Testimonials for "GET.ON" (IG2).	Testimonials for "StudiCare" (IG3).
**Fictitious testimonials based on a pilot study. Information: Name and age.**	**Self-constructed based on real testimonials for employees. Information: name, age, profession, and type of training.**	**Self-constructed based on real testimonials for students. Information: name, age, study subject, and type of training.**
"This internet-based therapy has really helped me. The training program and the contact with my therapist worked well." (Petra Z., 35)	"More and more, the stress I was experiencing at work was spilling over into my private life. Upon learning recurrent rules about coping with stress, I began to get used to applying them, and it became easier to deal with stress without building up additional stress in doing so." (Klaus M., 51, call center employee, stress management training)	"I was able to participate where and when it was good for me. It was this kind of flexibility that allowed me to commit to such a training while juggling my distance-learning studies, family life and job simultaneously - and it worked! Highly recommended!" (Claudia W, Psychology major, 37, stress management training)
"My experience was positive. I would recommend MH-Online to others." (Sebastian L., 46)	"It really helps me to write down some of my thoughts and receive your comments on them. Your answers give me the courage to work on myself and my problems and to get a handle on these problems. And they also give me some new perspectives by mentioning certain aspects or reflecting on situations in a different light. This in turn helps me reflect differently upon things." (Janine T., teacher, 52, prevention training for exhaustive depression)	"At first I was a bit surprised about the whole process, but very quickly I began to see similar advantages in this type of training as in my distance-learning studies. This is a wonderful first step into therapy and training. It helped me face the stress and the corresponding problems caused by the doubled strain of working and studying simultaneously. For me this was the first step towards getting help via face-to-face therapy. Without this "easy" kick-off, I would never have gone." (Tim K., Philosophy major, 40, prevention training for exhaustive depression)
"I could easily incorporate my MH-Online therapy sessions into my daily routine. My therapist was very responsive and supportive of me." (Jens R., 25)	"Prior to the training program, I was just running in circles trying to deal with my problems at work. I can only recommend this type of training to anyone who wants to work on themselves and needs to learn the right tools to do so. It helped me become much stronger in only a matter of weeks. Thank you for this! Sandra F., 46, staff member, problem-solving training)	"It is wonderful for me as a student of a distance-learning university to receive support for dealing with mental problems. I struggle specifically with test anxiety. The StudiCare training program and the respective feedback from my coach helped a lot with this. Just knowing that there is someone out there whom I can contact anonymously for help is such a relief. (Anna S., B.A. Communications, 29, test anxiety training)

S1 File. Supporting information.

Full overview of information texts with or without testimonials

For the sake of completeness, a differentiated full overview of the text-based information with or without testimonials per group is shown. This includes information that may be redundant in terms of identical contents that were already fully or partly described in the prior section.

Information intervention for the active control group (information only)

The following text will provide you with information on guided electronic mental health trainings and therapies to help cope with psychological problems such as stress and to build resilience. Please read the description carefully and answer the questions regarding your opinion on these novel interventions afterwards. There will be no questions to test your knowledge of these interventions.

Electronic mental health trainings and therapies with regards to mental health offer new opportunities to improve and/or expand care for afflicted persons. Mental illness often remains untreated. Internet-based psychotherapy can be considered a means to bridge the gap and offer care to more individuals. Numerous scientific studies have shown the efficacy of internet-based therapy for a broad range of psychological problems that can, for instance, arise from prolonged exposure to stress.

These internet-based interventions employ methods from Cognitive Behavioral Therapy that are also used in regular face-to-face therapy sessions. For instance, there are trainings to generally help cope with stress, to build resilience, to cope with mild or moderate depression, anxiety disorders or eating disorders. One of the currently existing forms are internet-based guided programs that allow patient to access online programs or online self-learning resources with the help of a therapist. Daily use of an app can also be employed. Patients communicate with their therapist via e-mail, chat or video conference. Flexibility regarding time and location is seen as a big advantage.

Information and untargeted testimonials in IG1 (hypothetical MH-Online)

The following text will provide you with information on guided electronic mental health trainings and therapies to help cope with psychological problems such as stress and to build resilience. Please read the description carefully and answer the questions regarding your opinion on these novel interventions afterwards. There will be no questions to test your knowledge of these interventions.

Electronic mental health trainings and therapies with regards to mental health offer new opportunities to improve and/or expand care for afflicted persons. Mental illness often remains untreated. Internet-based psychotherapy can be considered a means to bridge the gap and offer care to more individuals. Numerous scientific studies have shown the efficacy of internet-based therapy for a broad range of psychological problems that can, for instance, arise from prolonged exposure to stress.

These internet-based interventions employ methods from Cognitive Behavioral Therapy that

are also used in regular face-to-face therapy sessions. For instance, there are trainings to generally help cope with stress, to build resilience, to cope with mild or moderate depression, anxiety disorders or eating disorders. One of the currently existing forms are internet-based guided programs that allow patient to access online programs or online self-learning resources with the help of a therapist. Daily use of an app can also be employed. Patients communicate with their therapist via e-mail, chat or video conference. Flexibility regarding time and location is seen as a big advantage.

MH-Online (Albert-Ludwigs-Universität Freiburg) offers evidence-based and professionally guided internet-based training programs in areas such as “stress”, “resilience”, and “depression/anxiety”.

These online trainings consist of 6 consecutive one-hour sessions. You can accompany the online sessions with an app that allows for daily use. Additionally, you may receive personal coaching sessions. The training program aims to help you regain productivity. The resilience training program includes sessions on self-efficacy, optimism, relationships, and self-care. During the stress management training programs, participants learn to identify personal issues, practice coping mechanisms, apply problem-solving techniques, and learn how to manage difficult emotions.

"This internet-based therapy has really helped me. The training program and the contact with my therapist worked well." (Petra Z., 35)

“My experience was positive. I would recommend MH-Online to others.“ (Sebastian L., 46)

“I could easily incorporate my MH-Online therapy sessions into my daily routine. My therapist was very responsive and supportive of me.“ (Jens R., 25)

Information and testimonials targeted to employees in IG2 (GET.ON)

The following text will provide you with information on guided electronic mental health trainings and therapies to help cope with psychological problems such as stress and to build resilience. Please read the description carefully and answer the questions regarding your opinion on these novel interventions afterwards. There will be no questions to test your knowledge of these interventions.

Electronic mental health trainings and therapies with regards to mental health offer new opportunities to improve and/or expand care for afflicted persons. Mental illness often remains untreated. Internet-based psychotherapy can be considered a means to bridge the gap and offer care to more individuals. Numerous scientific studies have shown the efficacy of internet-based therapy for a broad range of psychological problems that can, for instance, arise from prolonged exposure to stress.

These internet-based interventions employ methods from Cognitive Behavioral Therapy that are also used in regular face-to-face therapy sessions. For instance, there are trainings to generally help cope with stress, to build resilience, to cope with mild or moderate depression, anxiety disorders or eating disorders. One of the currently existing forms are internet-based guided programs that allow patient to access online programs or online self-learning resources with the help of a therapist. Daily use of an app can also be employed. Patients communicate with their therapist via e-mail, chat or video conference. Flexibility regarding time and location

is seen as a big advantage.

The GET.ON Institute (Leuphana Universität Lüneburg) offers evidence-based and professionally guided internet-based training programs in areas such as “stress”, “resilience”, and “depression/anxiety”. Participation is open for various social groups, e.g. employed persons, trainees, students, retired persons.

These online trainings consist of 6 consecutive one-hour sessions. You can accompany the online sessions with an app that allows for daily use. Additionally, you may receive personal coaching sessions. The training program aims to help you regain productivity. The resilience training program includes sessions on self-efficacy, optimism, relationships, and self-care. During the stress management training programs, participants learn to identify personal issues, practice coping mechanisms, apply problem-solving techniques, and learn how to manage difficult emotions.

"More and more, the stress I was experiencing at work was spilling over into my private life. Upon learning recurrent rules about coping with stress, I began to get used to applying them, and it became easier to deal with stress without building up additional stress in doing so." (Klaus M., 51, call center employee, stress management training)

"Prior to the training program, I was just running in circles trying to deal with my problems at work. I can only recommend this type of training to anyone who wants to work on themselves and needs to learn the right tools to do so. It helped me become much stronger in only a matter of weeks. Thank you for this! Sandra F., 46, staff member, problem-solving training)

"It really helps me to write down some of my thoughts and receive your comments on them. Your answers give me the courage to work on myself and my problems and to get a handle on these problems. And they also give me some new perspectives by mentioning certain aspects or reflecting on situations in a different light. This in turn helps me reflect differently upon things." (Janine T., teacher, 52, prevention training for exhaustive depression)

Information and testimonials targeted to students in IG3 (StudiCare)

The following text will provide you with information on guided electronic mental health trainings and therapies to help cope with psychological problems such as stress and to build resilience. Please read the description carefully and answer the questions regarding your opinion on these novel interventions afterwards. There will be no questions to test your knowledge of these interventions.

Electronic mental health trainings and therapies with regards to mental health offer new opportunities to improve and/or expand care for afflicted persons. Mental illness often remains untreated. Internet-based psychotherapy can be considered a means to bridge the gap and offer care to more individuals. Numerous scientific studies have shown the efficacy of internet-based therapy for a broad range of psychological problems that can, for instance, arise from prolonged exposure to stress.

These internet-based interventions employ methods from Cognitive Behavioral Therapy that

are also used in regular face-to-face therapy sessions. For instance, there are trainings to generally help cope with stress, to build resilience, to cope with mild or moderate depression, anxiety disorders or eating disorders. One of the currently existing forms are internet-based guided programs that allow patient to access online programs or online self-learning resources with the help of a therapist. Daily use of an app can also be employed. Patients communicate with their therapist via e-mail, chat or video conference. Flexibility regarding time and location is seen as a big advantage.

StudiCare (Friedrich-Alexander-Universität Erlangen) offers evidence-based and professionally guided internet-based training programs in areas such as “stress”, “resilience”, and “depression/anxiety”.

These online trainings consist of 6 consecutive one-hour sessions. You can accompany the online sessions with an app that allows for daily use. Additionally, you may receive personal coaching sessions. The training program aims to help you regain productivity. The resilience training program includes sessions on self-efficacy, optimism, relationships, and self-care. During the stress management training programs, participants learn to identify personal issues, practice coping mechanisms, apply problem-solving techniques, and learn how to manage difficult emotions.

“It is wonderful for me as a student of a distance-learning university to receive support for dealing with mental problems. I struggle specifically with test anxiety. The StudiCare training program and the respective feedback from my coach helped a lot with this. Just knowing that there is someone out there whom I can contact anonymously for help is such a relief. (Anna S., B.A. Communications, 29, test anxiety training)

“At first I was a bit surprised about the whole process, but very quickly I began to see similar advantages in this type of training as in my distance-learning studies. This is a wonderful first step into therapy and training. It helped me face the stress and the corresponding problems caused by the doubled strain of working and studying simultaneously. For me this was the first step towards getting help via face-to-face therapy. Without this “easy“ kick-off, I would never have gone.“ (Tim K., Philosophy major, 40, prevention training for exhaustive depression)

“I was able to participate where and when it was good for me. It was this kind of flexibility that allowed me to commit to such a training while juggling my distance-learning studies, family life and job simultaneously - and it worked! Highly recommended!“ (Claudia W, Psychology major, 37, stress management training)

S1 File. Appendix 1 on the Methods section.

Appendix 1B. Outcome Measures

This section provides an overview of self-report measures in terms of e-mental-health- and stress-related items and scales, sorted by time point (translated from German to English). The original German questionnaire can be found in the GESIS repository (https://doi.org/10.7802/2127).

BASELINE ASSESSMENT

Awareness of and Experience with eMHS (AE, adapted from prior work [1])

AE. Had you heard about e-mental health services prior to this study? (*filter question*)

- If "yes" or "not sure" (*filter question*):

AE1. Have you ever gathered information about one or more e-mental health services?

AE2. Have you ever used one or more e-mental health services?

Baseline - Attitude towards eMHSs - short scale (BA, adapted from prior work, e.g. [1])

BA1. I consider e-mental health services to be generally useful.

BA2. I believe e-mental health services can be a sensible supplement to conventional health care services

BA3. I would recommend e-mental health services to friends, acquaintances or family members in case of stress, psychological problems or to build resilience.

- *Note*. Scale range from 1 ("totally disagree") to 7 ("totally agree").

Baseline - Intention to Use eMHSs (BI, i.e. acceptance; adapted from prior work [2]); methods and results on acceptance outcomes are reported elsewhere [3,4])

BI1. I can imagine that e-mental health services could increase my well-being.

BI2. I would like to try an e-mental health service.

BI3. I am planning to use an e-mental health service in the next 3 months.

- *Note*. Scale range from 1 ("totally disagree") to 7 ("totally agree").

Perceived Stress Scale (PSS-10); 10 items, reported elsewhere [5]

POST-INTERVENTION ASSESSMENT

Source credibility (SC, self-constructed)

SC1. Regarding the information presented, it was generally clear whether it was facts or opinions. *(Information credibility, all groups*)

SC2. Regarding the statements of the testimonials, it was generally clear whether they were facts or opinions. (*Testimonial credibility, IGs only)*

SC3. I found the testimonials' statements credible. (*Testimonial credibility, IGs only*)

SC4. I found the information presented to be credible. (*Information credibility, all groups*)

- *Note*. Two items/statements were shown to all four study arms (SC1, SC4), while the IGs received two additional items (SC2, SC3) on the testimonials´ credibility. *Note*. scale range 1 (“totally disagree”) to 7 (“totally agree”).

Perceived Similarity (PS, self-constructed)

PS1. These persons’ background is similar to mine.

PS2. These persons and I have a similar educational background.

PS3. These persons face similar strain as I do.

PS4. I feel affected in a similar way as these persons.

PS5. These persons‘ lives are similar to mine.

- *Note.* Perceived similarity was only assessed in the IGs (i.e. the three experimental study arms that were provided with testimonials in addition to general information). Scale range from1 (“totally disagree”) to 7 (“totally agree”).

Attitude – Post-intervention (AP, adapted from prior work, e.g. [1]), version for the CG

AP1. E-mental health services can be useful in coping with psychological problems or stress, and to build resilience.

AP2. I would recommend e-mental health services to my friends/acquaintances who are facing psychological problems or stress.

- *Note*. Scale range from 1 (“totally disagree”) to 7 (“totally agree”)

Intention to Use (i.e. acceptance) – Post-intervention (IP, adapted from prior work, [2]), version shown to the CG; methods and results on acceptance outcomes are reported elsewhere [3,4]

IP1. I can imagine that e-mental health services could increase my well-being.

IP2. I would like to try one of these services.

IP3. I plan to use such a service within the next three months.

- *Note*. Items were slightly modified for the IGs, with the presented eMHS as example, e.g. "I would like to try a service like StudiCare."). Scale range from 1 ("totally disagree") to 7 ("totally agree").

Attitude towards Psychological Online Interventions (APOI); 16-items, reported elsewhere [6]

Attitude towards internet-based therapies, e-therapy attitudes measure (ETAM); 17-items, German version [1]

ETAM1. Internet-based therapies are modern and in line with our modern times.

ETAM2. Internet-based therapies will replace conventional face-to-face psychotherapy in the future.

ETAM3. Internet-based therapy is more compatible with work and private life than conventional face-to-face therapy.

ETAM4. It makes no difference to me whether psychotherapy is conducted through the internet or in a psychotherapy practice in a clinic.

ETAM5. Internet-based therapies will reach more individuals with mental health problems.

ETAM6. Health insurance companies should cover the costs for internet-based therapies.

ETAM7. Internet-based therapy programs are as effective as conventional face-to-face psychotherapies.

ETAM8. Trust in a therapist can be just as easily built on the internet as in conventional face-to- face psychotherapy.

ETAM9. Internet-based therapies are an appropriate alternative to conventional face-to-face psychotherapy.

ETAM10. In case of mental health problems, I would attend an internet-based therapy.

ETAM11. I would prefer an internet-based therapy to a conventional face-to-face psychotherapy.

ETAM12. Internet-based therapies will reach more patients and help them.

ETAM13. I´m not particularly worried about data security in internet therapies.

ETAM14. The anonymity in internet therapies decreases the threshold to speak openly and honestly about important issues.

ETAM15. Through the dissemination of internet therapies, persons will get professional help earlier.

ETAM16. Misunderstandings occur in internet therapies as often as in conventional psychotherapies.

ETAM17. Internet therapies are suitable for most patients, regardless of their personal background (age, sex, education, etc.).

- *Note*. Scale range from 1 ("totally disagree") to 5 ("totally agree")

1 References

1. Apolinário-Hagen J, Harrer M, Kählke F, Fritsche L, Salewski C, Ebert DD. Public Attitudes Toward Guided Internet-Based Therapies: Web-Based Survey Study. JMIR Ment Health. 2018; 5: e10735. doi: 10.2196/10735.
2. Hennemann S, Beutel ME, Zwerenz R. Drivers and Barriers to Acceptance of Web-Based Aftercare of Patients in Inpatient Routine Care. J Med Internet Res. 2016; 18: e337. doi: 10.2196/jmir.6003.
3. Apolinário-Hagen J, Harrer M, Dederichs M, Fritsche L, Wopperer J, Wals F, et al. Effects of Narrative information to Increase the Acceptance of E-Mental Health Interventions among University Students: Four-Group Randomized Controlled Trial (Preprint); 2020.
4. Wopperer J, Apolinário-Hagen J, Wals F, Harrer, Kemper, J, Salewski C, Lehr D, et al. Exploring the usefulness of testimonials as a tool to improve the acceptance of e-mental health interventions among university students: preliminary results of a pilot RCT. 6th esrii Conference. Copenhagen, Denmark.
5. Klein EM, Brähler E, Dreier M, Reinecke L, Müller KW, Schmutzer G, et al. The German version of the Perceived Stress Scale – psychometric characteristics in a representative German community sample. BMC psychiatry. 2016; 16. doi: 10.1186/s12888-016-0875-9.
6. Schröder J, Sautier L, Kriston L, Berger T, Meyer B, Späth C, et al. Development of a questionnaire measuring Attitudes towards Psychological Online Interventions-the APOI. Journal of affective disorders. 2015; 187: 136–141. doi: 10.1016/j.jad.2015.08.044.
