# Supplementary material for: Exploring the influence of testimonial source on attitudes towards e-mental health interventions among university students: Four-group randomized controlled trial
Source: PLoS One. 2021 May 26;16(5):e0252012. doi: 10.1371/journal.pone.0252012 (PMC8153476; doi:10.1371/journal.pone.0252012)
Supplement: S2 File — Supplementary material on the results section. (PDF) [file pone.0252012.s002.pdf]

Supporting Information

S2 File. Appendix 2 on the Results section.

Supplementary analysis regarding the regression analysis.

Notes: Detailed information on the study data, including the data sets of the final (n=451) and the intention-to-treat (n=482) analyses, can be found here: GESIS repository (German “Leibniz Institut für Sozialwissenschaften”; SowiDataNet – datatorium); DOI: https://doi.org/10.7802/2127).

Supplementary Table S1. Sample characteristics in the intention-to-treat analysis

**Variables**		**N = 482**
**Gender**	Female, n (%)	359 (74.5)
	Male, n (%)	121 (25.1)
	Other/diverse, n (%)	1 (0.2)
**Age (years)**	Mean (SD),<br>median, range (years)	32.3 (10.15),<br>29.00, 18-65 years
**Educational attainment[a]**	General certificate of secondary school, n (%)	14 (2.9)
	Master craftsman´s diploma, n (%)	10 (2.1)
	Advanced college entrance qualification, n (%)	30 (6.2)
	University entrance qualification, n (%)	207 (42.9)
	College or university degree, n (%)	182 (37.8)
	- Bachelor degree, n (%)	99 (20.5)
	- Master degree, n (%)	61 (12.7)
	- PhD level, n (%)	22 (4.6)
	Other, n (%)	39 (8.1)
**Study program**	Distance-learning university, n (%)	426 (88.4)
	Traditional (on-campus) college or university, n (%)	30 (6.2)
	Distance-learning and traditional study program, n (%)	25 (5.2)
	Postgraduate program, n (%)	1 (0.2)
**Study time model**	Studying in full-time, n (%)	257 (53.3)

S2 File. Supporting information.

	Studying in part-time, n (%)	225 (46.7)
	Missing (postgraduate program), n (%)	1 (0.2)
**Awareness of e-mental health services**[b]	No	282 (48.5)
	Yes	228 (39.2)
	Not sure	48 (8.3)

N=482.

[a]Educational attainment refers to the German education system.

[b]Question: “Had you heard about e-mental health services prior to this study?”

As shown in Table S2, post hoc tests indicated that source credibility was higher in IG2 with *GET.ON* testimonials for employees than in IG1 with untargeted “MH-Online” testimonials. These differences were found overall (Games-Howell, $mean_{diff}$=0.42, *SE*=0.13, *p*=0.005, 95% CI [0.76, 0.09]; *d*=0.45), as well as for information alone (Tuckey-HSD, $mean_{diff}$=0.38, *SE*=0.14, *p*=0.021, 95% CI [0.03, 0.76]; *d*=0.38) and testimonials alone (Tuckey-HSD, $mean_{diff}$=0.45, *SE*=0.13, *p*=0.002, 95% CI [0.13, 0.77]; *d*=0.43). The three IGs (n=339) differed significantly regarding the perceived similarity with the testimonial personas, with a large ES ($ŋ_p^2$=.15, *d*=0.84). Games-Howell-adjusted post hoc tests showed that participants in IG3 (*StudiCare*) perceived narrators as significantly more similar to themselves as those in IG1 on the fictitious “MH-Online” ($mean_{diff}$=0.67, *SE*=0.14, *p*<0.001, 95% CI [0.36, 0.98]; *d*=0.67) and in IG3 on *GET.ON* ($mean_{diff}$=1.05, *SE*=0.15, *p*<0.001, 95% CI [0.71, 1.40]; *d*=0.93). Perceived similarity was also assessed higher in IG1 (fictitious “MH-Online”, with untargeted testimonials) than in IG2 (*GET.ON*), with testimonials targeting employees ($mean_{diff}$=0.39, *SE*=0.14, *p*=0.013, 95% CI [0.07, 0.71]; *d*=0.38).

Supplementary Table S2. Descriptive data and between-group differences regarding the determinants of attitudes towards e-mental health services among university students.

Scale / Construct	Range scale (min-max)	Overall (n=451) M (SD)	CG (n=112) M (SD)	IG 1 MH-Online (n=115) M (SD)	IG 2 GET.ON (n=116) M (SD)	IG 3 StudiCare (n=108) M (SD)	Difference[a] (groups), effect size	Post-hoc tests[b,c]
Perceived Stress – PSS-10 (10 items), mean	1-5 (1.20-4.70)	2.71 (0.66)	2.70 (0.66)	2.69 (0.71)	2.70 (0.62)	2.76 (0.65)	$F_{(3,447)}$ = 0.24, p=0.865, η_p^2=.002	N.A.
Perceived Stress – PSS-10 (10 items), sum score	10-50 (12-47)	27.04 (6.64)	26.88 (6.64)	26.88 (7.15)	26.91 (6.28)	27.53 (6.54)	$F_{(3,447)}$ = 0.25, p=0.860, η_p^2=.002	N.A.
Perceived Similarity (5 items, IGs only, n=339)	1-7 (1.00-7.00)	3.95 (1.12) (n=339)	N.A. in CG	3.87 (0.89)	3.48 (1.15)	4.54 (1.12)	$F_{(2,336)}$ = 29.21, p<0.001, η_p^2= .15 (large ES)	In IG3 higher than in IG1 and IG2, and in IG1 than IG2[b] (*ps*<0.05)
Source Credibility (4 items, overall)[d]	1-7 (1.00-7.00)	5.05 (1.00)	5.15 (1.14)	4.79 (1.07)	5.22 (0.85)	5.06 (0.88)	$F_{(3,447)}$ = 4.07, p=0.007, η_p^2=.03 (small ES)	In IG2 (GET.ON) higher than in IG1[b] (p=0.005)
Source Credibility (2 items, information credibility)[d]	1-7 (1.00-7.00)	5.08 (1.05)	5.15 (1.14)	4.86 (1.13)	5.25 (0.91)	5.07 (0.98)	$F_{(3,447)}$ = 2.99, p=0.031, η_p^2=.02 (small ES)	IG2 higher than IG1[c], (p=0.021)
Source Credibility (2 items, testimonial credibility, IG1-IG3, n=339)[d]	1-7 (1.00-7.00)	4.98 (1.02) (n=339)	N.A.[d]	4.73 (1.12)	5.18 (0.95)	5.05 (0.93)	$F_{(3,336)}$ = 6.12, p=0.002, η_p^2=.04 (small ES)	In IG2 higher than in IG1[c], (p=0.002), in IG3 higher than in IG1[c] (p=0.002),

Notes. N=451. Abbreviations: ES=effect size, N.A.=not applicable.

[a]One-way ANOVA

[b]Post hoc test with Games-Howell adjustment

[c]Post hoc test with Tuckey-HSD-adjustment

[d]Perceived credibility was assessed with two items in the CG (in the IGs with four items).

S2 File. Supporting information.

Supplementary Table S3. Hierarchical multiple regression of information type, perceived stress, perceived similarity and source credibility on attitudes towards e-mental health services for stress coping (intention-to-treat analysis).

**Step 1**	**Variable**	**B**	***SE* (B)**	**ß**	**T**	***p*-value**	**95% CI (B)**	
	(Constant)	.60	0.32		1.88	0.060	-0.03	1.21
	IG3 vs. IG2	.24	0.13	.08	1.85	0.065	-0.02	0.49
	IG3 vs. IG1	.27	0.12	.09	2.14	0.033	0.02	0.51
	Stress Level	.07	0.07	.04	1.08	0.281	-0.06	0.21
	Perceived Similarity	.24	0.05	.20	4.96	<0.001	0.15	0.34
R^2=.37	Source Credibility	.63	0.04	.56	15.10	<0.001	0.55	0.71
**Step 2**	**Variable**	**B**	***SE* (B)**	**ß**	**T**	***p*-value**	**95% CI (B)**	
	(Constant)	-.47	0.25		-1.88	0.061	-0.97	0.02
	IG3 vs. IG2	.01	0.10	.004	0.12	0.907	-0.19	0.21
	IG3 vs. IG1	.20	0.10	.07	2.07	0.039	0.01	0.39
	Stress Level	.08	0.05	.04	1.50	0.135	-0.03	0.19
	Perceived Similarity	.12	0.04	.10	3.00	0.003	0.04	0.19
	Source Credibility	.35	0.04	.31	9.92	<0.001	0.28	0.42
R^2=.63	Baseline Attitude	.60	0.03	.58	17.94	<0.001	0.54	0.67

N=482. Dependent variable: Attitude towards eMHSs at post-intervention; IG1=testimonials for MH-Online, IG2=testimonials for GET.ON, IG3=testimonials for StudiCare, *SE*=standard error B, CI=confidence interval. B=unstandardized coefficient; β=standardized coefficient (beta-weight). Step 1: R^2=.37, $F_{(5,476)}$ = 56.97, p<0.001, (adjusted R^2=.37); step 2: R^2=.63, $F_{(1,475)}$ = 321.74, p<0.001 (adjusted R^2=.62), increase ΔR^2 =.25.

Values of the CG for items on perceived similarity and credibility of testimonials were imputed (mean imputation).

Results of the regression analyses using listwise deletion (excl. the CG) for the per protocol (n=339) and ITT (n=361) sample, can be found elsewhere (SPSS output files, in the GESIS repository).

Output files on the mediation analyses using the PROCESS macro

Notes: Variable names in the output files were modified for reporting purposes, since two researchers independently conducted the analyses with the same original data set, but used slightly different label names for the translation from German to English. The final material can be found here: https://doi.org/10.7802/2127.

Per protocol analysis

Attitude short scale (post-intervention)

- Dependent variable (Y): Attitude toward eMHSs for stress coping (post-intervention)
- Independent variable (X): Targeted information type (student vs. employee)
- Mediator (Z): Perceived similarity

```
Run MATRIX procedure:

**************** PROCESS Procedure for SPSS Version 3.00 *****************

          Written by Andrew F. Hayes, Ph.D.       www.afhayes.com
    Documentation available in Hayes (2018). www.guilford.com/p/hayes3

**************************************************************************
Model  : 4
    Y  : mean_att (mean attitude short scale)
    X  : study_ar (study arm_IG2vsIG3; dummy-coded)
    M  : mean_sim (mean similarity)

Sample
Size:  224

**************************************************************************
OUTCOME VARIABLE:
 mean_sim

Model Summary
          R       R-sq        MSE          F        df1        df2          p
      ,4308      ,1856     1,2284    50,5941     1,0000   222,0000      ,0000

Model
              coeff         se          t          p       LLCI       ULCI
constant     3,4810      ,1029    33,8273      ,0000     3,2782     3,6838
study_ar     1,0542      ,1482     7,1130      ,0000      ,7621     1,3462

**************************************************************************
OUTCOME VARIABLE:
 mean_att
```

```
Model Summary
          R       R-sq        MSE          F        df1        df2          p
      ,3716      ,1381      ,9436    17,7043     2,0000   221,0000      ,0000

Model
              coeff         se          t          p       LLCI       ULCI
constant     4,0243      ,2238    17,9855      ,0000     3,5834     4,4653
study_ar     -,4423      ,1439    -3,0728      ,0024     -,7259     -,1586
mean_sim      ,3484      ,0588     5,9224      ,0000      ,2325      ,4643

************************** TOTAL EFFECT MODEL ****************************
OUTCOME VARIABLE:
 mean_att

Model Summary
          R       R-sq        MSE          F        df1        df2          p
      ,0361      ,0013     1,0885      ,2893     1,0000   222,0000      ,5912

Model
              coeff         se          t          p       LLCI       ULCI
constant     5,2371      ,0969    54,0636      ,0000     5,0462     5,4280
study_ar     -,0750      ,1395     -,5378      ,5912     -,3500      ,1999

************** TOTAL, DIRECT, AND INDIRECT EFFECTS OF X ON Y **************

Total effect of X on Y
     Effect         se          t          p       LLCI       ULCI       c_ps
     -,0750      ,1395     -,5378      ,5912     -,3500      ,1999     -,0720

Direct effect of X on Y
     Effect         se          t          p       LLCI       ULCI      c'_ps
     -,4423      ,1439    -3,0728      ,0024     -,7259     -,1586     -,4246

Indirect effect(s) of X on Y:
            Effect     BootSE   BootLLCI   BootULCI
mean_sim      ,3672      ,0791      ,2237      ,5343

Partially standardized indirect effect(s) of X on Y:
            Effect     BootSE   BootLLCI   BootULCI
mean_sim      ,3526      ,0701      ,2228      ,4989

*********************** ANALYSIS NOTES AND ERRORS ************************

Level of confidence for all confidence intervals in output:
  95,0000

Number of bootstrap samples for percentile bootstrap confidence intervals:
  5000

NOTE: Variables names longer than eight characters can produce incorrect output.
      Shorter variable names are recommended.

------ END MATRIX -----
```

Attitude toward online therapies (ETAM)

- Dependent variable (Y): Attitude toward online therapies, ETAM (post-intervention)
- Independent variable (X): Targeted information type (student vs. employee)
- Mediator (Z): Perceived similarity

```
Run MATRIX procedure:

**************** PROCESS Procedure for SPSS Version 3.00 *****************

          Written by Andrew F. Hayes, Ph.D.       www.afhayes.com
    Documentation available in Hayes (2018). www.guilford.com/p/hayes3

**************************************************************************
Model  : 4
    Y  : mean_eta (mean etam)
    X  : study_ar (study arm_IG2vsIG3; dummy-coded)
    M  : mean_sim (mean similarity)

Sample
Size:  224

**************************************************************************
OUTCOME VARIABLE:
 mean_sim

Model Summary
          R       R-sq        MSE          F        df1        df2          p
      ,4308      ,1856     1,2284    50,5941     1,0000   222,0000      ,0000

Model
              coeff         se          t          p       LLCI       ULCI
constant     3,4810      ,1029    33,8273      ,0000     3,2782     3,6838
study_ar     1,0542      ,1482     7,1130      ,0000      ,7621     1,3462

**************************************************************************
OUTCOME VARIABLE:
 mean_eta

Model Summary
          R       R-sq        MSE          F        df1        df2          p
      ,2104      ,0443      ,2458     5,1207     2,0000   221,0000      ,0067

Model
              coeff         se          t          p       LLCI       ULCI
constant     2,8350      ,1142    24,8245      ,0000     2,6100     3,0601
study_ar     -,1535      ,0735    -2,0895      ,0378     -,2983     -,0087
mean_sim      ,0927      ,0300     3,0876      ,0023      ,0335      ,1519

************************** TOTAL EFFECT MODEL ****************************
OUTCOME VARIABLE:
 mean_eta

Model Summary
```

S2 File. Supporting information.

```
         R       R-sq        MSE          F        df1        df2          p
     ,0553      ,0031      ,2553      ,6818     1,0000   222,0000      ,4098

Model
              coeff         se          t          p       LLCI       ULCI
constant     3,1577      ,0469    67,3135      ,0000     3,0653     3,2502
study_ar     -,0558      ,0676     -,8257      ,4098     -,1889      ,0774
```

```
************** TOTAL, DIRECT, AND INDIRECT EFFECTS OF X ON Y **************

Total effect of X on Y
     Effect         se          t          p       LLCI       ULCI       c_ps
     -,0558      ,0676     -,8257      ,4098     -,1889      ,0774     -,1105

Direct effect of X on Y
     Effect         se          t          p       LLCI       ULCI      c'_ps
     -,1535      ,0735    -2,0895      ,0378     -,2983     -,0087     -,3040

Indirect effect(s) of X on Y:
             Effect     BootSE   BootLLCI   BootULCI
mean_sim      ,0977      ,0327      ,0375      ,1652

Partially standardized indirect effect(s) of X on Y:
             Effect     BootSE   BootLLCI   BootULCI
mean_sim      ,1936      ,0634      ,0748      ,3209
```

```
********************** ANALYSIS NOTES AND ERRORS ************************

Level of confidence for all confidence intervals in output:
  95,0000

Number of bootstrap samples for percentile bootstrap confidence intervals:
  5000

NOTE: Variables names longer than eight characters can produce incorrect output.
      Shorter variable names are recommended.

------ END MATRIX -----
```

Attitudes toward psychological online interventions (APOI)

- Dependent variable (Y): Attitude toward online therapies/interventions, APOI (post-intervention)
- Independent variable (X): Targeted information type (student vs. employee)
- Mediator (Z): Perceived similarity

```
Run MATRIX procedure:

**************** PROCESS Procedure for SPSS Version 3.00 *****************

          Written by Andrew F. Hayes, Ph.D.       www.afhayes.com
    Documentation available in Hayes (2018). www.guilford.com/p/hayes3

**************************************************************************
Model  : 4
    Y  : mean_apo (mean apoi)
    X  : study_ar (study arm_IG2vsIG3; dummy-coded)
    M  : mean_sim (mean similarity)

Sample
Size:  224

**************************************************************************
OUTCOME VARIABLE:
 mean_sim

Model Summary
          R       R-sq        MSE          F        df1        df2          p
      ,4308      ,1856     1,2284    50,5941     1,0000   222,0000      ,0000

Model
              coeff         se          t          p       LLCI       ULCI
constant     3,4810      ,1029    33,8273      ,0000     3,2782     3,6838
study_ar     1,0542      ,1482     7,1130      ,0000      ,7621     1,3462

Covariance matrix of regression parameter estimates:
           constant   study_ar
constant      ,0106     -,0106
study_ar     -,0106      ,0220

**************************************************************************
OUTCOME VARIABLE:
 nean_apo

Model Summary
          R       R-sq        MSE          F        df1        df2          p
      ,2325      ,0541      ,2133     6,3150     2,0000   221,0000      ,0022

Model
              coeff         se          t          p       LLCI       ULCI
constant     2,8604      ,1064    26,8902      ,0000     2,6508     3,0700
study_ar     -,1053      ,0684    -1,5388      ,1253     -,2402      ,0296
```

```
mean_sim      ,0994      ,0280     3,5539      ,0005      ,0443      ,1545

Covariance matrix of regression parameter estimates:
           constant   study_ar   mean_sim
constant      ,0113      ,0010     -,0027
study_ar      ,0010      ,0047     -,0008
mean_sim     -,0027     -,0008      ,0008

************************** TOTAL EFFECT MODEL ****************************
OUTCOME VARIABLE:
 mean_apoi

Model Summary
          R       R-sq        MSE          F        df1        df2          p
      ,0006      ,0000      ,2244      ,0001     1,0000   222,0000      ,9933

Model
              coeff         se          t          p       LLCI       ULCI
constant     3,2064      ,0440    72,8933      ,0000     3,1197     3,2930
study_ar     -,0005      ,0633     -,0084      ,9933     -,1254      ,1243

Covariance matrix of regression parameter estimates:
           constant   study_ar
constant      ,0019     -,0019
study_ar     -,0019      ,0040
```

```
************** TOTAL, DIRECT, AND INDIRECT EFFECTS OF X ON Y **************

Total effect of X on Y
     Effect         se          t          p       LLCI       ULCI       c_ps
     -,0005      ,0633     -,0084      ,9933     -,1254      ,1243     -,0011

Direct effect of X on Y
     Effect         se          t          p       LLCI       ULCI      c'_ps
     -,1053      ,0684    -1,5388      ,1253     -,2402      ,0296     -,2228

Indirect effect(s) of X on Y:
             Effect     BootSE   BootLLCI   BootULCI
mean_sim      ,1048      ,0330      ,0447      ,1731

Partially standardized indirect effect(s) of X on Y:
             Effect     BootSE   BootLLCI   BootULCI
mean_sim      ,2216      ,0681      ,0963      ,3604
```

```
*********************** ANALYSIS NOTES AND ERRORS ************************

Level of confidence for all confidence intervals in output:
  95,0000

Number of bootstrap samples for percentile bootstrap confidence intervals:
  5000

NOTE: Variables names longer than eight characters can produce incorrect output.
      Shorter variable names are recommended.

------ END MATRIX -----
```

Intention-to-treat mediation analyses

```
Run MATRIX procedure:

**************** PROCESS Procedure for SPSS Version 3.00 *****************

          Written by Andrew F. Hayes, Ph.D.       www.afhayes.com
    Documentation available in Hayes (2018). www.guilford.com/p/hayes3

**************************************************************************
Model  : 4
    Y  : S_Attitu = mean_att (mean attitude short scale)
    X  : IG3vsIG2 = study_ar (study arm_IG2vsIG3; dummy-coded)
    M  : S_Simila = mean_sim (mean similarity)

Sample
Size:  241

**************************************************************************
OUTCOME VARIABLE:
 S_Simila

Model Summary
          R       R-sq        MSE          F        df1        df2          p
      ,3861      ,1491     1,2877    41,8785     1,0000   239,0000      ,0000

Model
              coeff         se          t          p       LLCI       ULCI
constant     3,5317      ,1023    34,5174      ,0000     3,3301     3,7333
IG3vsIG2      ,9463      ,1462     6,4714      ,0000      ,6582     1,2343

**************************************************************************
OUTCOME VARIABLE:
 S_Attitu

Model Summary
          R       R-sq        MSE          F        df1        df2          p
      ,4006      ,1605     1,0636    22,7471     2,0000   238,0000      ,0000

Model
              coeff         se          t          p       LLCI       ULCI
constant     3,7853      ,2275    16,6391      ,0000     3,3371     4,2334
IG3vsIG2     -,4835      ,1441    -3,3564      ,0009     -,7673     -,1997
S_Simila      ,3934      ,0588     6,6928      ,0000      ,2776      ,5093

************************** TOTAL EFFECT MODEL ****************************
OUTCOME VARIABLE:
 S_Attitu

Model Summary
          R       R-sq        MSE          F        df1        df2          p
      ,0497      ,0025     1,2584      ,5922     1,0000   239,0000      ,4423

Model
              coeff         se          t          p       LLCI       ULCI
constant     5,1748      ,1011    51,1601      ,0000     4,9755     5,3741
```

```
IG3vsIG2     -,1112      ,1446     -,7695      ,4423     -,3960      ,1735

************** TOTAL, DIRECT, AND INDIRECT EFFECTS OF X ON Y **************

Total effect of X on Y
     Effect         se          t          p       LLCI       ULCI       c_ps
     -,1112      ,1446     -,7695      ,4423     -,3960      ,1735     -,0992

Direct effect of X on Y
     Effect         se          t          p       LLCI       ULCI      c'_ps
     -,4835      ,1441    -3,3564      ,0009     -,7673     -,1997     -,4314

Indirect effect(s) of X on Y:
             Effect     BootSE   BootLLCI   BootULCI
S_Simila      ,3723      ,0832      ,2217      ,5533

Partially standardized indirect effect(s) of X on Y:
             Effect     BootSE   BootLLCI   BootULCI
S_Simila      ,3322      ,0679      ,2065      ,4724

*********************** ANALYSIS NOTES AND ERRORS ************************

Level of confidence for all confidence intervals in output:
  95,0000

Number of bootstrap samples for percentile bootstrap confidence intervals:
  5000

NOTE: Variables names longer than eight characters can produce incorrect output.
      Shorter variable names are recommended.

------ END MATRIX -----




Run MATRIX procedure:

**************** PROCESS Procedure for SPSS Version 3.00 *****************

          Written by Andrew F. Hayes, Ph.D.       www.afhayes.com
    Documentation available in Hayes (2018). www.guilford.com/p/hayes3

**************************************************************************
Model  : 4
    Y  : S_ETAM = mean_eta (mean ETAM)
    X  : IG3vsIG2 = study_ar (study arm_IG2vsIG3; dummy-coded)
    M  : S_Simila = mean_sim (mean similarity)

Sample
Size:  241

**************************************************************************
OUTCOME VARIABLE:
 S_Simila
```

```
Model Summary
          R       R-sq        MSE          F        df1        df2          p
      ,3861      ,1491     1,2877    41,8785     1,0000   239,0000      ,0000

Model
              coeff         se          t          p       LLCI       ULCI
constant     3,5317      ,1023    34,5174      ,0000     3,3301     3,7333
IG3vsIG2      ,9463      ,1462     6,4714      ,0000      ,6582     1,2343

**************************************************************************
OUTCOME VARIABLE:
 S_ETAM

Model Summary
          R       R-sq        MSE          F        df1        df2          p
      ,2481      ,0616      ,2553     7,8075     2,0000   238,0000      ,0005

Model
              coeff         se          t          p       LLCI       ULCI
constant     2,7413      ,1115    24,5929      ,0000     2,5217     2,9609
IG3vsIG2     -,1450      ,0706    -2,0547      ,0410     -,2841     -,0060
S_Simila      ,1125      ,0288     3,9070      ,0001      ,0558      ,1693

************************** TOTAL EFFECT MODEL ****************************
OUTCOME VARIABLE:
 S_ETAM

Model Summary
          R       R-sq        MSE          F        df1        df2          p
      ,0372      ,0014      ,2706      ,3307     1,0000   239,0000      ,5658

Model
              coeff         se          t          p       LLCI       ULCI
constant     3,1388      ,0469    66,9207      ,0000     3,0464     3,2312
IG3vsIG2     -,0385      ,0670     -,5751      ,5658     -,1706      ,0935

************** TOTAL, DIRECT, AND INDIRECT EFFECTS OF X ON Y **************

Total effect of X on Y
     Effect         se          t          p       LLCI       ULCI       c_ps
     -,0385      ,0670     -,5751      ,5658     -,1706      ,0935     -,0742

Direct effect of X on Y
     Effect         se          t          p       LLCI       ULCI      c'_ps
     -,1450      ,0706    -2,0547      ,0410     -,2841     -,0060     -,2792

Indirect effect(s) of X on Y:
             Effect     BootSE   BootLLCI   BootULCI
S_Simila      ,1065      ,0347      ,0439      ,1797

Partially standardized indirect effect(s) of X on Y:
             Effect     BootSE   BootLLCI   BootULCI
S_Simila      ,2050      ,0625      ,0871      ,3327

*********************** ANALYSIS NOTES AND ERRORS ************************

Level of confidence for all confidence intervals in output:
```

```
  95,0000

Number of bootstrap samples for percentile bootstrap confidence intervals:
  5000

NOTE: Variables names longer than eight characters can produce incorrect output.
      Shorter variable names are recommended.

------ END MATRIX -----



Run MATRIX procedure:

**************** PROCESS Procedure for SPSS Version 3.00 *****************

          Written by Andrew F. Hayes, Ph.D.       www.afhayes.com
    Documentation available in Hayes (2018). www.guilford.com/p/hayes3

**************************************************************************
Model  : 4
    Y  : S_APOI = mean_apo (mean APOI)
    X  : IG3vsIG2 = study_ar (study arm_IG2vsIG3; dummy-coded)
    M  : S_Simila = mean_sim (mean similarity)

Sample
Size:  241

**************************************************************************
OUTCOME VARIABLE:
 S_Simila

Model Summary
          R       R-sq        MSE          F        df1        df2          p
      ,3861      ,1491     1,2877    41,8785     1,0000   239,0000      ,0000

Model
              coeff         se          t          p       LLCI       ULCI
constant     3,5317      ,1023    34,5174      ,0000     3,3301     3,7333
IG3vsIG2      ,9463      ,1462     6,4714      ,0000      ,6582     1,2343

**************************************************************************
OUTCOME VARIABLE:
 S_APOI

Model Summary
          R       R-sq        MSE          F        df1        df2          p
      ,2460      ,0605      ,2036     7,6672     2,0000   238,0000      ,0006

Model
              coeff         se          t          p       LLCI       ULCI
constant     2,8515      ,0995    28,6471      ,0000     2,6554     3,0476
IG3vsIG2    -,1052      ,0630    -1,6695      ,0963     -,2294      ,0189
S_Simila      ,1006      ,0257     3,9121      ,0001      ,0500      ,1513

************************** TOTAL EFFECT MODEL ****************************
OUTCOME VARIABLE:
 S_APOI
```

```
Model Summary
          R       R-sq        MSE          F        df1        df2          p
      ,0108      ,0001      ,2158      ,0280     1,0000   239,0000      ,8672

Model
              coeff         se          t          p       LLCI       ULCI
constant     3,2069      ,0419    76,5607      ,0000     3,1244     3,2894
IG3vsIG2     -,0100      ,0599     -,1674      ,8672     -,1279      ,1079
```

**************** TOTAL, DIRECT, AND INDIRECT EFFECTS OF X ON Y **************

```
Total effect of X on Y
     Effect         se          t          p       LLCI       ULCI       c_ps
     -,0100      ,0599     -,1674      ,8672     -,1279      ,1079     -,0216

Direct effect of X on Y
     Effect         se          t          p       LLCI       ULCI      c'_ps
     -,1052      ,0630    -1,6695      ,0963     -,2294      ,0189     -,2270

Indirect effect(s) of X on Y:
             Effect     BootSE   BootLLCI   BootULCI
S_Simila      ,0952      ,0277      ,0445      ,1514

Partially standardized indirect effect(s) of X on Y:
             Effect     BootSE   BootLLCI   BootULCI
S_Simila      ,2054      ,0578      ,0982      ,3230

*********************** ANALYSIS NOTES AND ERRORS ************************

Level of confidence for all confidence intervals in output:
  95,0000

Number of bootstrap samples for percentile bootstrap confidence intervals:
  5000

NOTE: Variables names longer than eight characters can produce incorrect output.
      Shorter variable names are recommended.

------ END MATRIX -----
```
