# Supplementary material for: Exploring the influence of testimonial source on attitudes towards e-mental health interventions among university students: Four-group randomized controlled trial
Source: PLoS One. 2021 May 26;16(5):e0252012. doi: 10.1371/journal.pone.0252012 (PMC8153476; doi:10.1371/journal.pone.0252012)
Supplement: S3 File — CONSORT-PSI 2018 checklist. (PDF) [file pone.0252012.s003.pdf]

S3 File. Appendix 3. (submitted version of the manuscript)

CONSORT-SPI 2018 Checklist

SECTION	ITEM #	CONSORT-SPI 2010	CONSORT-SPI 2018	REPORTED ON PAGE #
**TITLE AND ABSTRACT**				
	1a	Identification as a randomised trial in the title§		1
	1b	Structured summary of trial design, methods, results, and conclusions (for specific guidance see CONSORT for Abstracts)§	Refer to CONSORT extension for social and psychological intervention trial abstracts	2
**INTRODUCTION**				
Background and Objectives	2a	Scientific background and explanation of rationale §		3
	2b	Specific objectives or hypotheses §	If pre-specified, how the intervention was hypothesied to work	7
**METHODS**				
Trial Design	3a	Describe of trial design (such as parallel, factorial), including allocation ratio §	If the unit of random assignment is not the individual, please refer to CONSORT for Cluster Randomized Trials	8
	3b	Important changes to methods after trial commencement (such as eligibility criteria), with reasons		NA
Participants	4a	Eligibility criteria for participants§	When applicable, eligibility criteria for settings and those delivering the interventions	9
	4b	Settings and locations where the data were collected		8,9
Interventions	5	The interventions for each group with sufficient details to allow replication, including how and when they are actually administered §		11
	5a		Extent to which interventions were actually delivered by providers and taken up by participants as planned	NA

	5b		Where other informational materials about delivering the intervention can be accessed	NA
	5c		When applicable, how intervention providers were assigned to each group	NA
Outcomes	6a	Completely defined pre-specified outcomes, including how and when they were assessed§		13
	6b	Any changes to trial outcomes after the trial commenced, with reasons		NA
Sample Size	7a	How sample size was determined§		9
	7b	When applicable, explanation of any interim analyses and stopping guidelines		9
**RANDOMISATION**				
Sequence generation	8a	Method used to generate the random allocation sequence		10
	8b	Type of randomisation; detail of any restriction (such as blocking and block size)§		10
Allocation concealment mechanism	9	Mechanism used to implement the random allocation sequence, describing any steps taken to conceal the sequence until interventions were assigned§		10
Implementation	10	Who generated the random allocation sequence, who enrolled participants, and who assigned participants to interventions§		10
Awareness of assignment	11a	Who was aware of intervention assignment after allocation (for example, participants, providers, those assessing outcomes), and how any masking was done		10
	11b	If relevant, description of the similarity of interventions		11, 12

Analytical methods	12a	Statistical methods used to compare group outcomes§	How missing data were handled, with details of any imputation method	15-17
	12b	Methods for additional analyses, such as subgroup analyses, adjusted analyses, and process evaluations		16
**RESULTS**				
Participant flow (a diagram is strongly recommended)	13a	For each group, the numbers randomly assigned, receiving the intended intervention, and analysed for the outcomes§	Where possible, the number approached, screened, and eligible prior to random assignment, with reasons for non-enrolment	17, Fig 2
	13b	For each group, losses and exclusions after randomisation, together with reasons§		17, Fig 2
Recruitment	14a	Dates defining the periods of recruitment and follow-up		9
	14b	Why the trial ended or was stopped		NA
Baseline data	15	A table showing baseline characteristics for each group§	Include socioeconomic variables where applicable	18
Numbers analysed	16	For each group, number included in each analysis and whether the analysis was by original assigned groups§		18
Outcomes and estimation	17a	For each outcome, results for each group, and the estimated effect size and its precision (such as 95% confidence interval)§	Indicate availability of trial data	
	17b	For binary outcomes, the presentation of both absolute and relative effect sizes is recommended		
Ancillary analyses	18	Results of any other analyses performed, including subgroup analyses, adjusted analyses, and process evaluations, distinguishing pre-specified from exploratory		
Harms	19	All important harms or unintended effects in each group (for specific guidance see CONSORT for Harms)		
**DISCUSSION**				

Limitations	20	Summarize the main results (including an overview of concepts, themes, and types of evidence available), link to the review questions and objectives, and consider the relevance to key groups.	Trial limitations, addressing sources of potential bias, imprecision, and, if relevant, multiplicity of analyses	24, 28
Generalisability	21	Discuss the limitations of the scoping review process.	Generalisability (external validity, applicability) of the trial findings§	28
Interpretation	22	Provide a general interpretation of the results with respect to the review questions and objectives, as well as potential implications and/or next steps.	Interpretation consistent with results, balancing benefits and harms, and considering other relevant evidence	24-27
**IMPORTANT INFORMATION**				
Registration	23	Registration number and name of trial registry		NA
Protocol	24	Where the full trial protocol can be accessed, if available		NA
Declaration of Interests	25	Sources of funding and other support; role of funders	Declaration of any other potential interests	30
Stakeholder investments	26a		Any involvement of the intervention developer in the design, conduct, analysis, or reporting of the trial	NA
	26b		Other stakeholder involvement in trial design, conduct, or analyses	NA
	26c		Incentives offered as part of the trial	9

This table lists items from the CONSORT 2010 checklist (with some modifications for social and psychological intervention trials) and additional items in the CONSORT-SPI 2018 extension. Empty rows in the 'CONSORT-SPI 2018' column indicate that there is no extension to the CONSORT 2010 item

*We strongly recommended that the CONSORT-SPI 2018 Explanation and Elaboration (E&E) document be reviewed when using the CONSORT-SPI 2018 checklist for important clarifications on each item

§An extension item for cluster trials exists for this CONSORT 2010 item

Note: Fig 2 (participant flow chart) is Fig3 in the revised manuscript.

Citations

Montgomery, P., Grant, S., Mayo-Wilson, E., Macdonald, G., Michie, S., Hopewell, S., & Moher, D. (2018). Reporting randomised trials of social and psychological interventions: the CONSORT-SPI 2018 Extension. *Trials*, *19*(1), 407.

Grant, S., Mayo-Wilson, E., Montgomery, P., Macdonald, G., Michie, S., Hopewell, S., & Moher, D. (2018). CONSORT-SPI 2018 Explanation and Elaboration: guidance for reporting social and psychological intervention trials. *Trials*, *19*(1), 406.
